# Supplementary material for: Determinants and pathways of healthcare-seeking behaviours in under-5 children for common childhood illnesses and antibiotic prescribing: a cohort study in rural India
Source: BMJ Open. 2021 Dec 2;11(12):e052435. doi: 10.1136/bmjopen-2021-052435 (PMC8647549; doi:10.1136/bmjopen-2021-052435)

Additional File 1: Socio-demographic survey questionnaire

Part1: Information on household members:

S.No.	Name	Sex (M/F)	Age	Relation with 'Head'	Educational Qualification	Occupation
1.						
2.						
3.						
4.						
5.						
6.						
7.						
8.						
9.						
10.						

Part2: Questionnaire

Gender of head of household
Respondent age
Gender of respondent
Family type
Number of family members
Number of children of age 0 to 1 year, 1 to 3 years, 0 to 5 years, 6 to 15 years
Number of members above 15 years of age
Type of caste
Which ration card does the family have
Type of house (kuccha, semi-kuccha, pucca)?
Do you own your house?
Number of rooms other than kitchen, toilet, storage
Do you pay bill for electricity
First priority source of electricity used
Second priority source of electricity used
Third priority source of electricity used
Fourth priority source of electricity used
Does your household possess:

- Radio
- Television
- Bicycle
- Motor cycle
- Bull/horse
- Car/jeep
- Tractor
- Harvester
- Generator

LPG
Water pump in house
water pump in field
Landline (Phone)
Mobile (Phone)
Internet
Cooler, table fan or Ceiling fan
Fridge
Diesel Engine
Livestock
Do you own any property and agriculture land?
If yes - How much property other than agriculture in square feet (sq.ft)?
If yes - How much is for agriculture in sq.ft?
First priority main source of drinking water
What kind of toilet facility do members of your household usually use?

Additional File 2: Healthcare-seeking behavior diary

S.NO.	QUESTIONS
	**Section A: General information about illness**
1.	History of presenting illness
2.	Did you seek any treatment for your child or gave anything on your own to your child for the illness?
3.	If **'NO'**, Why? **(end the interview)**
4.	If **'YES'**, Where?
	**Section B: Treatment at "HOME"**
5.	If 'HOME', describe the treatment? **(if 'NO' skip to 12)**
6.	What medicine you gave your child? mention the type, dose and source (**ask for**
7.	Who advice you the above mentioned treatment for your child's illness?
8.	From where did you get the medicine **(over the counter etc)?**
9.	Estimated direct cost of home treatment **(in rupees)**
10.	Did your child got relief from the illness **(YES / NO)**
11.	If **'NO'**, where did you took your child next for the treatment
12.	Name of '**Healthcare facility/private practitioner/CHV/ LHV/Pharmacist** etc.' visited for
13.	Where the healthcare facility/ clinic are located?
14.	Why you took your child to this healthcare facility/ private practitioner?
15.	Mode of transport to reach the facility?
16.	Travel time **(in minutes)**
17.	Did they prescribe any medicine?
18.	If **'YES'** describe the medicine with the respective dose /provide prescription?
19.	Did you give your child the complete dose of prescribed medicine? **(YES/NO)** **(if 'YES' skip to 21)**
20.	Why you did not gave the prescribed dose to your child?
21	Were there any adverse events from any of the medicines prescribed?
22.	Did your child have any relief from the illness? **(YES / NO)**
23.	Were your child asked for any investigation? **(YES/NO)** AndIf**"YES"** mention the type of investigation? **(if 'NO' skip to 25)**
24.	Did you take your child for the investigations? **(YES/NO)**
25.	Estimated direct cost of treatment from healthcare facility / private practitioner **(excluding the travel cost) (in rupees)**
26.	Transport Cost to reach healthcare facility/ private practitioner
	**Section D: Treatment by "Traditional healers"**
27.	Did you seek any treatment from the Traditional healers for your child's illness? **(YES/NO)**
28.	What kind of treatment was provided by them? Describe treatment
29.	Did your child got any relief from the illness? **(YES/NO)**
30.	Where do you generally seek the treatment for your child when he/she gets common cold, cough, fever, body ache?

Additional File 3: Presenting illness classification

**Presenting illness classification**	**Key illnesses under presenting illness classification**
RTI*	Cold,cough with/without fever,ear infection, tonsillitis
GI infections*	Diarrhoea with/without stomach pain, appetite loss vomiting, gas, constipation
Fever	Fever without other illness
Others	Illnesses that occurred rarely e.g. chicken pox, ring worm, injury, insect/animal bite, oral problems

* RTI- Acute respiratory tract infections, GI infections- Gastrointestinal infections

Additional File 4: Flow chart describing sample population, episodes of illness recorded among study population, under study in rural Ujjain, India.

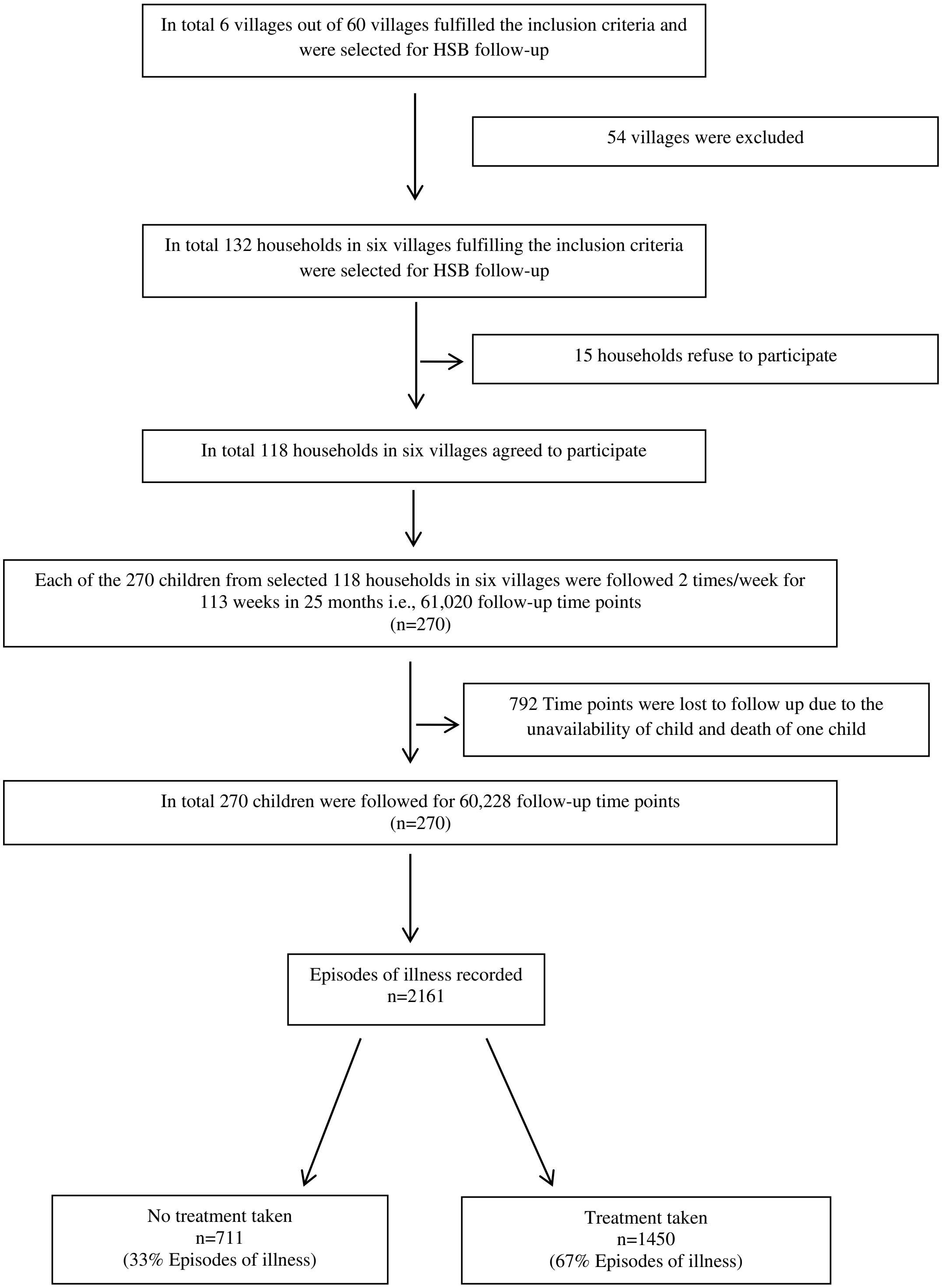

Supplement: Supplementary data [file bmjopen-2021-052435supp001.pdf]
