# Supplementary material for: Determinants and pathways of healthcare-seeking behaviours in under-5 children for common childhood illnesses and antibiotic prescribing: a cohort study in rural India
Source: BMJ Open. 2021 Dec 2;11(12):e052435. doi: 10.1136/bmjopen-2021-052435 (PMC8647549; doi:10.1136/bmjopen-2021-052435)
Supplement: Author's manuscript [file bmjopen-2021-052435.draft_revisions.pdf]

BMJ Open is committed to open peer review. As part of this commitment we make the peer review history of every article we publish publicly available.

When an article is published we post the peer reviewers' comments and the authors' responses online. We also post the versions of the paper that were used during peer review. These are the versions that the peer review comments apply to.

The versions of the paper that follow are the versions that were submitted during the peer review process. They are not the versions of record or the final published versions. They should not be cited or distributed as the published version of this manuscript.

BMJ Open is an open access journal and the full, final, typeset and author-corrected version of record of the manuscript is available on our site with no access controls, subscription charges or pay-per-view fees (http://bmjopen.bmj.com).

If you have any questions on BMJ Open's open peer review process please email info.bmjopen@bmj.com

BMJ Open

Determinants and pathways of healthcare-seeking behaviours in under-5 children for common childhood illnesses and antibiotic prescribing: A cohort study in rural India

Journal:	*BMJ Open*
Manuscript ID	bmjopen-2021-052435
Article Type:	Original research
Date Submitted by the Author:	19-Apr-2021

Complete List of Authors:	khare, shweta; Karolinska Institute, Department of Global Public Health ; Ruxmaniben Deepchand Gardi Medical College, Department of Public Health and Environment<br>Pathak, Ashish; Karolinska Institute, Department of Global Public Health ; Ruxmaniben Deepchand Gardi Medical College, Department of Pediatrics<br>Purohit, Manju; Karolinska Institute, Department of Global Public Health ; Ruxmaniben Deepchand Gardi Medical College, Department of Pathology<br>Sharma, Megha; Karolinska Institutet, Department of Global Public Health; RD Gardi Medical College, Department of Pharmacology<br>Marrone, Gaetano; Karolinska Institutet, Global Health (IHCAR)/Department of Global Public Health<br>Tamhankar, Ashok ; Karolinska Institute, Department of Global Public Health; Ruxmaniben Deepchand Gardi Medical College, Department of Environmental Medicine<br>Lundborg, Cecilia; Karolinska Institutet, Department of Global Public Health<br>Diwan, Vishal; Karolinska Institutet, Department of Global Public Health; ICMR- National Institute for Research in Environment Health

Keywords:	Quality in health care < HEALTH SERVICES ADMINISTRATION & MANAGEMENT, Public health < INFECTIOUS DISEASES, Community child health < PAEDIATRICS, PRIMARY CARE, PUBLIC HEALTH

SCHOLARONE™
Manuscripts

I, the Submitting Author has the right to grant and does grant on behalf of all authors of the Work (as defined in the below author licence), an exclusive licence and/or a non-exclusive licence for contributions from authors who are: i) UK Crown employees; ii) where BMJ has agreed a CC-BY licence shall apply, and/or iii) in accordance with the terms applicable for US Federal Government officers or employees acting as part of their official duties; on a worldwide, perpetual, irrevocable, royalty-free basis to BMJ Publishing Group Ltd ("BMJ") its licensees and where the relevant Journal is co-owned by BMJ to the co-owners of the Journal, to publish the Work in this journal and any other BMJ products and to exploit all rights, as set out in our licence.

The Submitting Author accepts and understands that any supply made under these terms is made by BMJ to the Submitting Author unless you are acting as an employee on behalf of your employer or a postgraduate student of an affiliated institution which is paying any applicable article publishing charge ("APC") for Open Access articles. Where the Submitting Author wishes to make the Work available on an Open Access basis (and intends to pay the relevant APC), the terms of reuse of such Open Access shall be governed by a Creative Commons licence – details of these licences and which Creative Commons licence will apply to this Work are set out in our licence referred to above.

Other than as permitted in any relevant BMJ Author's Self Archiving Policies, I confirm this Work has not been accepted for publication elsewhere, is not being considered for publication elsewhere and does not duplicate material already published. I confirm all authors consent to publication of this Work and authorise the granting of this licence.

For peer review only

Determinants and pathways of healthcare-seeking behaviours in under-5 children for common childhood illnesses and antibiotic prescribing: A cohort study in rural India

Shweta Khare[1,2#], Ashish Pathak[1,4,5], Manju Purohit[1,6], Megha Sharma[1,7], Gaetano Marrone[1], Ashok J Tamhankar[1,8], Cecilia Stålsby Lundborg[1], Vishal Diwan[1,3]

[1]Department of Global Public Health, Health Systems and Policy: Medicines focusing antibiotics, Karolinska Institutet, Tomtebodavagen18A, SE-171 77 Stockholm, Sweden

[2]Department of Public Health and Environment, RD Gardi Medical College, Ujjain, 456006, Madhya Pradesh, India

[3]ICMR- National Institute for Research in Environment Health, Bhopal, 462030, Madhya Pradesh, India

[4]Department of Pediatrics, RD Gardi Medical College, Ujjain, 456006, Madhya Pradesh, India

[5]Department of Women and Children's Health, International Maternal and Child Health Unit, Uppsala University, Uppsala SE-751 85, Sweden

[6]Department of Pathology, RD Gardi Medical College, Ujjain, 456006, Madhya Pradesh, India

[7]Department of Pharmacology, RD Gardi Medical College, Ujjain, 456006, Madhya Pradesh, India

[8]Indian Initiative for Management of Antibiotic Resistance, Department of Environmental Medicine, RD Gardi Medical College, Ujjain, 456006, Madhya Pradesh, India

[#]Corresponding author email: shweta.khare@ki.se; contact number: +91-9893986241

KEYWORDS: Healthcare-seeking behavior, Healthcare-seeking pathways, Common childhood illness, Carergivers, Under-5 children, Antibiotic prescribing, Rural population, India

WORD COUNT: 4840

REFERENCE COUNT: 49

ABSTRACT

Objectives

To explore the healthcare-seeking pathways, antibiotic prescribing and determine the socio-demographic factors associated with HSB of caregivers for common illnesses in under-5(U-5) children in rural Ujjain, India.

Study design Prospective cohort study.

Study setting and study sample

The cohort included 270 U-5 children from selected six villages in rural demographic surveillance site, of the R.D.Gardi Medical College, Ujjain, Madhya Pradesh, India. A community-based cohort was visited twice weekly for over 113 weeks(August 2014-October 2016) to record the HSB of caregivers using HSB diaries. Socio-demographic information was also solicited.

Primary and secondary outcome measures

Primary outcomes: First point of care, healthcare-seeking pathway and quantify antibiotic prescribing for the common acute illnesses.

Secondary outcome: HSB risk factors determined using mixed-effects multinomial logistic regression

Results

A total of 60,228 HSB follow-up time points for 270 children were recorded with a total of 2,161 acute illness episodes. The commonest illnesses found were RTI(69%)andGI(8%). No healthcare was sought in 33% of illness episodes, mostly for RTIs. The commonest healthcare-seeking pathway was to informal healthcare providers(IHCPs,49% of illness episodes). The adjusted relative risk(aRR) for obtaining no treatment, home treatment and treatment by IHCPs was higher for RTIs(aRR=11.54,1.82and1.29 respectively), illiterate mothers(aRR=2.86,2.38and1.93 respectively), and mothers who were homemakers(aRR=2.90,4.17and2.10 respectively). Socio-economic status was associated with HSB, with the highest aRR for no treatment in the lowest two socio-economic quintiles(aRR=6.59and6.39 respectively). Antibiotics were prescribed in 46%(n=670/1450) illness episodes and the majority (85%,n=572/670) were broad spectrum.

Conclusion

In our rural cohort for many acute episodes of illnesses no treatment or home treatment was done, which resulted in overall reduced antibiotic prescribing. The commonest healthcare-seeking pathway was to visit IHCPs, which indicates that they are major healthcare providers in rural areas. Most of the antibiotics were prescribed by IHCPs and were commonly prescribed for illnesses where they were not indicated.

ARTICLE SUMMARY

Strengths and limitations

- This is the largest ever healthcare-seeking behavior study to prospectively follow the healthcare-seeking behaviour of caregivers of a cohort of U-5 children treating common childhood illnesses and the antibiotic prescribing within a rural community setting in India.
- The longitudinal follow-up with repeated recordings of the healthcare-seeking behaviour occasions from the same cohort, made the data reliable.
- The study determined the pathway in healthcare-seeking behaviour for acute childhood illnesses, which have not been reported in studies from India.
- Information on the episodes of illness were subjective in nature and were not validated externally by any medical examination.

For peer review only - http://bmjopen.bmj.com/site/about/guidelines.xhtml

INTRODUCTION

Resistance to antibiotics has become a major threat to global health [1]. The crisis of antibiotic resistance compromises the ability to treat bacterial infections, which were once thought to have been contained [2]. Antibiotics are inappropriately used in humans, in animals as growth promoters, and also in aquaculture [3]. However, the greatest concern is for human use as this affects us directly.

Globally, 5.4 million children die before the age of 5 years per year, and about 50% of these deaths are caused by infectious diseases [4]. The commonest infections in children includes acute respiratory tract infections (RTIs) and acute diarrhoea, both of which are more often a result of viral infections, for which antibiotic treatment is not recommended [5-7]. However, antibiotics are often prescribed incorrectly and for un-indicated conditions, for example in viral infections [8-10]. Higher rates of antibiotic prescribing are associated with higher rates of antibiotic resistance among the bacterial pathogens that cause common infections [11].

India has one of the largest three-tier healthcare system (primary, secondary, tertiary) in the world. Each of the 3 levels of the heathcare system consist of both public and private healthcare providers which are a mix of allopathic and AYUSH (Ayurvedic, Unani, Siddha, Homeopathic - the non-allopathic systems of medicine practiced in India) practitioners. The practitioners of all the above systems are reported to be prescribing antbtioics. Antibiotics are also prescribed and/ or dispensed by the informal healthcare providers (IHCPs) and pharmacist [8, 12]. Antibiotics can also be purchased “over the counter” without prescription despite a law against it [8, 13]. Antibiotic prescribing rates in any community can be affected by the prescribing practices of healthcare providers, which are influenced by multiple factors [14-16] and the healthcare-seeking

behaviour (HSB) of the community. Studies have focused on how to influence the prescribing of antibiotics, but the factors affecting the HSB of a community have not been widely studied in regards to antibiotic prescribing. HSB includes any action or decision taken by a caregiver to regain the good health of a sick child [17]. In India, more than 60% of the population lives in rural areas [18], yet India's access to health care facilities is significantly urban biased [19]. Following the decision to seek healthcare, the subsequent pathway followed within the healthcare system often encompasses all available healthcare options, beginning with home care and traditional healers and extending to informal or formal health care services [20]. Gaining an understanding of the HSB of an individual is complex, and this behaviour is affected by multiple factors, including how medicine is practiced by existing healthcare providers, geographical location and socio-demographic factors [14-16]. These factors also influence antibiotic prescribing and are thus related to the development of antibiotic resistance [14-16].

Currently, there is limited literature on the determinants of HSB and antibiotic prescribing in rural areas, and furthermore, the pathway in HSB for acute childhood illnesses in India have not been studied. Therefore, the primary objective of the present prospective cohort study was to explore these healthcare-seeking pathways, investigate the HSB of caregivers and antibiotic prescribing for common acute infectious illnesses in under-5 (U-5) children. The secondary objective was to determine the socio-demographic factors associated with caregiver HSB following healthcare in the rural areas of Ujjain, India.

METHODS

Study design

Prospective cohort study with repeated follow-ups performed for 113 weeks from August 2014 to October 2016.

Study setting

This study was conducted in rural areas of the Ujjain district of Madhya Pradesh (MP) in central India. MP is India's second largest state by area and has 72 million inhabitants, with about 72% of the population residing in rural areas [21]. MP has poor health indicators (i.e., infant mortality rate of 51/1,000 live births, U-5 mortality rate of 65/1,000 live births [22]) and a human development index of 0.606 [23]. In this study, the rural demographic surveillance site (DSS) Palwa of Ruxmaniben Deepchand Gardi Medical College (RDGMC) was used. Six out of 60 villages were selected from this DSS, one of which was assigned as a central village. The six villages were selected using the following inclusion criteria: a) aerial distance of less than 5km from the central village, b) apopulation of at least 500 inhabitants c) and at least 15 children available in the desired age range of ≤5 years [24]. The map in Figure 1 shows the 5km study frame around the central village along with geographical location of the study villages, practising IHCPs and formal healthcare facilities. In addition, IHCPs and formal healthcare facilities at a 5km distance outside the study frame are also shown. Further details of the study settings are described elsewhere [24].

Study cohort

After selecting the 6 villages as described above all the households were listed. A total 132 households in the six villages satisfied the inclusion criteria: a) had childen U-5 and of having lived in the villages for the past one year, b) planned to live in the same village for the next two years and c) had parents consenting to participate in the study. Therefore, total of 118 households

having 270 U-5 children were included in the study. All children from a given family were included if eligible.

Sample size calculation

Estimates of proportions from a single batch of 100 observations (e.g., of children) have a precision of +/-10%. Using repeated batches of 100 children per batch (each week per month) has a power of 80% for detecting a linear trend for any observation in proportion of 0.1 per time unit (step between batches, negative or positive). A conservative estimate of a design effect of 2 was deemed appropriate in consideration of the differences in population sizes among the selected villages [7, 25], giving a required minimum sample size of (100×2) 200 children. The number of children included in the cohort was increased by 10% to accommodate for possible attrition over time. Thus, a minimum sample size of 220 children could provide a sufficiently high precision in the estimates and a power of at least 80% to the comparisons between data points.

Data collection

Healthcare-seeking behaviour follow-up

At baseline, the socio-demographic characteristics of all the selected families were collected through a questionnaire that surveyed household amenities and assets and family member education levels and occupations **(Additional File 1)**. HSB survey forms, called ‘healthcare-seeking behaviour diaries’, were designed to meet the study requirements and piloted and checked for face validity **(Additional File 2)**.

Furthermore, a pilot study was done to test the data collection instrument and associated procedures and to train and prepare the research assistants. No changes were deemed necessary in the data collection instrument after the pilot. In the main study, the HSBs of caregivers for acutely sick children were recorded twice a week by trained research assistants familiar with the local language. An acutely sick child was defined according to the perceptions of the caregiver. The classification of the sickness was performed by the research assistants and not by the caregiver. The follow-ups were completed using HSB diaries by the research assistants for 113 consecutive weeks between August 2014 and October 2016. An entry in the HSB diary was created for the duration of an illness irrespective of the fact that the illness may have started prior to the research assistant's last visit. One HSB diary was maintained per child, so if a household had more than one participating child, a corresponding number of diaries was maintained. The entries in the HSB diary included the relevant history related to an episode of illness, whether or not any home treatment or clinic or hospital-based treatment was provided, the child's route through different healthcare systems following an illness episode, the reasons for taking those routes when seeking healthcare, treatment details with a focus on any prescribed antibiotics and treatment costs and hospitalisations. The informal healthcare providers usually dispense medicines to the patient by themselves and so do not provide any written prescriptions. In cases where prescriptions were not available the caregivers were asked to retain the labelled wrappers/bottles of the medicines prescribed by the treating healthcare provider, and for the medicines which were dispensed loose, the details of such medicines and injections were asked from IHCPs. The entries in the HSB diaries were updated on subsequent biweekly visits until the episode of illness was resolved/cured or the caregiver stopped providing any treatment for that episode. Thus, one entry in the HSB diary was kept for each episode of illness per child. A new

For peer review only - http://bmjopen.bmj.com/site/about/guidelines.xhtml

entry in the HSB diary was made for new signs and symptoms or if old signs and symptoms developed again after one day of recovery. Diaries were checked and completed for missing information for an episode of illness twice weekly by the first author.

Data management

Independent variables

The independent variables for the study were: a) illnesses recorded during the HSB follow-up, classified as RTIs, gastrointestinal (GI) infections, fever, skin infections and others (details are presented in **Additional File 3**), b) age of child in years, c) age of caregiver in years as ≤25 years versus >25 years, d) education of mother as illiterate versus school education and higher (where school education included primary school education from class 1 to class 8 and secondary school education from class 9 to class 12); e) occupation of mother as a home maker versus working (including farmers, labourers, and public/private sector jobs); f) number of family members as ≤5 members versus >5 members, g) parity of mother categorised as 1 child, 2 children and ≥3 children and h) socio-economic status which was determined using the principal component analysis approach as referenced [26] and was categorized as: first quintile (poorest), second quintile (poorer), third quintile (middle), fourth quintile (wealthier), and fifth quintile (wealthiest).

Outcome variables

The primary outcomes were: 1) the first point of care for an episode of illness, 2) healthcare-seeking pathway that demonstrated the caregiver's route through different healthcare systems following an episode of illness in a child and 3) antibiotic prescribing for an episodes of illness

where some kind of treatment was provided to a sick child. The secondary outcomes were to determine the association of the socio-demographic factors related to the sick child and child's caregiver with the HSB.

Data analysis

Data were converted from the paper HSB diaries to an electronic format using Microsoft .NET Framework 4 (Microsoft Corp., Redmond, WA, USA). Stata 14.1 (Stata Corp. College Station, TX, USA) and IBM SPSS Statistics 23.0 (IBM Corp., Armonk, NY, USA) were used for data management and analysis.

Descriptive statistics was used to portray the demographic characteristics of the caregivers and children. The incidence of the different types of childhood illnesses and the HSB of the caregivers of the U-5 children for the recorded illnesses were calculated. Furthermore, the incidences of the different types of childhood illnesses were examined over four seasons. The seasons were divided as follows: pre-monsoon season (March to May), monsoon season (June to September), post-monsoon season (October to December) and winter season (January and February), which have average maximum temperatures of 37.5 ºC, 30 ºC, 29 ºC and 22 ºC, respectively [27]. The outcome of the analysis for the primary outcome was the first point of care for an episode of illness and the proportion of illness episodes receiving an antibiotic prescription and was not based on the individual child. The first point of care was the caregiver's HSB from the onset of an child's illness to the first healthcare service approached by the caregiver in seeking treatment for the illness. This HSB was characterized as: 1) no treatment given, 2) home treatment (i.e., episodes of illness for which caregivers provided children home-based treatments, leftover medicines or medicines from local shops without consulting recognised

For peer review only - http://bmjopen.bmj.com/site/about/guidelines.xhtml

healthcare providers), 3) healthcare-seeking from formal healthcare providers (i.e., episodes of illness where caregivers consulted formal healthcare practitioners, including medical practitioners recognized by the Indian Medical Council working in a government or private healthcare facility, community health visitors, lady health visitors or providers in health camps organised by medical institutes) [28] and 4) healthcare-seeking from IHCPs (i.e., episodes of illness where caregivers went to IHCPs for treatment, defined as healthcare providers who have not received a formal degree in medicine from any institution and who are not registered as healthcare practitioners by any governing body including informal private practitioners, local unlicensed pharmaceutical vendors and traditional healers) [29]. A mixed-effects logistic regression model accounting for two levels of clusters (children belonging to the same family and therefore having the same caregiver and the same children who had several episodes of a sickness during the follow-up period) was used to estimate the effects of children and caregiver characteristics on caregiver HSB over time. The percentage of illness episodes with an RTI (69%) was higher than that of the other common illnesses (GI infections-8%, fever-7%, skin infection-6% and others-10%) **(Table 1)**. Since, the number of episodes of RTI was greater than that of any other illness, the factors associated with the HSB of caregivers are presented only for RTI, whereas for all other complaints, the proportions of different HSBs are presented **(Table 1)**. The antibiotic prescription patterns for different episodes of illness were explored and analysed. Each prescribed antibiotic was coded according to the World Health Organization (WHO) Collaborating Centre for Drug Statistics Methodology, Anatomical Therapeutic Chemical (ATC) classification with the defined daily dose according to the fifth level of the ATC classification, J01 (antibacterial for systemic use) [30]. Antibiotic prescriptions were presented as the

proportion of illness episodes receiving an antibiotic prescription and the number of antibiotic courses. The frequency and percentage were calculated for the categorical variables.

A mixed-effects multinomial stepwise logistic regression model was used to examine the association of the independent risk factors responsible for HSB taking treatment from formal healthcare providers as the base outcome. The independent risk factors were compared between getting no treatment, receiving home treatment and obtaining treatment from IHCPs (multinomial outcome variable), and the adjusted relative risk (aRR) along with the associated 95% confidence intervals (CI) and *p* values were reported.

Ethical considerations

All methods were carried out in accordance with the ethical standards set by the institutional ethics committee. An oral informed consent was obtained from all participating caregivers and from parents or guardians on behalf of child participants in the local language, Hindi.

Participating parents/guardians were explained about the study purposes and procedures orally and textually in the local language (Hindi). Parents/guardians were informed that they could withdraw from the study at any time. The identities of participating parents/guardians and children were masked using unique identifiers designed for the study, and no private demographic information was shared publicly. Furthermore, all healthcare providers' data collected from parents/guardians was kept non-identifiable. Additional assistance was provided to children in need of medical attention during follow-up by referral to nearby formal health facilities. Health camps were also organised after the study for all the children living in the study villages.

For peer review only - http://bmjopen.bmj.com/site/about/guidelines.xhtml

Patient and public involvement

Patients and the public were not involved in the design or planning of the study.

RESULTS

Out of a total possible 61,020 follow-up time points of HSB for 270 children available throughout the study period, 792 time points were lost to follow up due to the unavailability of the children (travelling) and death of one child **(Additional File 4)**. So, the overall response rate for the study was 99%.

Socio-demographic characteristics of caregivers and children

Table 2 summarizes the socio-demographic characteristics of the children and their caregivers. The mean (±standard deviation [SD]) age of the children was 1.8±1.4years. All of the primary caregivers of the 270 children in the study were mothers. The mean (±SD) age of mothers was 24.8±5.21 years and ranged from 14 to 50 years. On average, each mother had two children. Half of the mothers had some school education, and 71% were homemakers. Of the mothers, 62% were from the households of families with more than five members and half were from households of poor socio-economic status.

Illness episodes, illness types and seasonal variations in the number of illness episodes

During the study period, a total of 2,161 illness episodes were recorded in the 270 children. The average (±SD) number of illness episodes recorded per child was 8±5 episodes, (range1 to 29). The average (±SD) duration for which an episode of illness persisted was 4.4±3.7 days, (range1 to 63 days). The commonest illness reported in the study was RTIs (69%, **Table 1**).

For peer review only - http://bmjopen.bmj.com/site/about/guidelines.xhtml

A peak in the number of illness episodes was observed in the winter season (January-February 2015) during the 26th week of follow-up, which recorded 41 illness episodes. Another peak was observed in the second post-monsoon season (October-December 2014) during the 65th week with 36 illness episodes **(Figure 2)**.

Healthcare-seeking behaviours of caregivers and the mapping of the healthcare services approached for the treatment of illnesses in children

The caregivers of the children sought care from two types of healthcare providers: IHCPs and formal healthcare providers, and they also preferred home care for a child's illness. For 33% of episodes (n = 711), no treatment was offered to the child, most commonly for RTI (n = 92%, 654/711). In 86% (n = 607/711) of the illness episodes where no care/treatment was sought, the mothers believed that the illnesses were mild and self-limiting **(Table 1)**.

Of the remaining episodes (n = 1450) in which some healthcare was provided for the illness, most mothers approached IHCPs for treatment, accounting for 73% (n = 1062/1450) of these cases. The distribution of the different types of IHCPs from where the treatments were sought was as follows: informal private practitioners (90%, n = 962/1062), local drug vendors (7%, n=78/1062) and traditional healers (3%, n =22/1062). Formal health care providers were approached for 5% (n = 72/1450) of illness episodes, and the different types included formal private practitioners (44%, n=32/72), public/private healthcare facilities (24%, n=17/72), lady health visitors (26%, n=19/72) and treatment providers at health camps conducted by medical colleges (6%, n=4/72). The second most common choice for child's illness was giving home care (22%, n=316/1,450; **Table 1** and **Figure 3**). Home care included providing medicines in 67% (n = 211/316, which included leftover medicines from

previous prescriptions and getting medicines prescribed in old prescription from local general/medical store) and homemade remedies in 35% (n = 105/316, which included herbal tea, herbal oil, herbal paste made of cloves and honey).

Of those illness episodes where care was sought, in 72% (n = 1045/1450) illness episodes mothers provided care on the same day as the start of the illness **(Table 1)**. The main reasons for a delay of more than 24 hours in seeking healthcare were: lack of money, lack of time and no transport facility available at the time of illness.

Healthcare-seeking pathways

A conceptual framework of the healthcare-seeking pathways was developed using the information from the HSB diaries. Figure 3 describes the overall healthcare-seeking pathways of the caregivers of the children during the episodes of illness irrespective of the type of illness. The dashed arrows in Figure3 show the HSB of the mothers from the onset of illness to the first point of care. The pathways demonstrate that IHCPswere the most preferred first point of care and accounted for 73% (n = 1062/1450) of the cases in which a child received treatment **(Figure3)**. The continuous arrows in Figure 3 represent the change in healthcare-seeking pathways between: home care, IHCPs and formal healthcare providers. Some caregivers appeared to switch between multiple healthcare facilities or providers (8%, n = 118/1450), especially when the illnesses lasted for more than one week. The most common pathway taken was to switch from one IHCP to another. In 4% (n = 52/1450) of the illness episodes regardless of the illness duration, treatment at home or by a traditional healer was performed along with allopathic therapy.

Mixed-effects multinomial logistic regression results for the effect of socio-demographic factors on the healthcare-seeking behaviour of caregivers

For peer review only - http://bmjopen.bmj.com/site/about/guidelines.xhtml

The risk factors for HSB were determined by mixed-effects multinomial logistic regression with treatment from formal healthcare providers as a base outcome, as seen in Table 3. The aRR for getting no treatment, receiving home care and obtaining treatment from IHCPs was higher when the type of illness was RTI as compared to the other illnesses reported (aRR = 11.54, 95% CI 6.14-21.71; 1.82, 95% CI 1.03-3.24 and 1.29, 95% CI 0.75-2.23 respectively), among illiterate mothers as compared to educated mothers (aRR = 2.86, 95% CI 1.41-5.80; 2.38, 95% CI 1.21-4.68 and 1.93, 95% CI 0.90-3.74 respectively), and when the mother was a home maker as compared to working mothers (aRR = 2.90, 95% CI 1.43-5.90; 4.17, 95% CI 2.06-8.46 and 2.10, 95% CI 1.16-3.79 respectively). Socio-economic status was associated with HSB across all quintiles, with the highest aRR for no treatment in the two lowest socio-economic quintiles (aRR = 6.59, 95% CI 2.15-20.23 and 6.39, 95% CI 2.13-18.86 respectively), as seen in Table 3.

Antibiotic prescribing for common childhood illnesses

Out of a total of 1450 illness episodes, 46% (n=670) received antibiotics. In a total of 670 episodes where antibiotics were given, a total of 807 antibiotic courses were prescribed and administered as single course of antibiotics (69%, n = 559/807) and up to a total of four antibiotics (14%, n = 111/807). In total, 85% (n = 694/807) of the administered antibiotic courses were prescribed by IHCPs. The majority (85%, n = 572/670) of antibiotics prescribed were broad spectrum and the most commonly prescribed were third-generation cephalosporin (J01DD) (n=259, 39%) followed by penicillin with an extended spectrum (J01CA) (n=94, 14%) and fluoroquinolones (J01MA) (n=86, 13%) **(Table 4)**. Antibiotics were prescribed more frequently in cases of skin infections (62%) and GI infections (45%) as compared to fever alone (35%) **(Table 1)**.

DISCUSSION

This is the first study to prospectively follow the HSB of caregivers of a cohort of U-5 children treating common childhood illnesses and the antibiotic prescribing during the treatment in a community setting over a period of 2 years. This study explored healthcare-seeking pathways and antibiotic prescribing for acute childhood illnesses and investigated the socio-demographic determinants of caregivers affecting the HSB during an event of acute illness in a child.

The findings highlight important information about rural HSB during common childhood illnesses in India. The healthcare-seeking pathway differed considerably among caregivers because of an interplay of a variety of factors. For example, India's wealthier classes tend to seek healthcare from multiple providers, whereas the poorer classes often get no treatment at all [19, 31]. The different points of care approached by the caregivers were: informal private practitioners, local drug vendors, traditional healers, formal private practitioners, public/private healthcare facilities, lady health visitors and providers of treatment at health camps conducted by medical colleges. Each caregiver visited at least one of the above listed healthcare service providers once or more for a variety of reasons during the study period. IHCPs were the most frequent service providers for first and second point of healthcare visits. Only 5% of the mothers took their child to formal healthcare providers. In our study informal private practitioners (66%) were the most greatly preferred choice of caregivers when seeking healthcare despite existing formal healthcare facilities in the nearby vicinity **(Figure 3)**. The result contrast with those of other studies from India that have reported that government facilities were approached more often [32, 33]. However, private providers were also found to be preferred in India's state of West Bengal [34]. Public and private healthcare systems coexist in almost all countries with limited resources. India has one of the most privatized health systems in the world [35]. The

388 reason why private healthcare providers are generally preferred is due to their ease of access and the inability of public healthcare systems to provide access to quality care [36]. Public hospitals in India have had issues related to low quality treatments, long waiting periods, inadequate infrastructure and doctor shortages [37]. Most administrative authorities are indifferent to educating IHCPs and encourage them to work illegally as IHCPs address the needs unmet by the formal healthcare system [38, 39].

394 In our study, RTIs were the most commonly occurring illnesses reported in U-5 children, which is unlike previous cross-sectional studies that have reported greater incidence of diarrhoeal cases as compared to respiratory infection [33]. This may be attributed to Swachh Bharat Mission (SBM)/Swachh Bharat Abhiyan (SBA)/Clean India Mission a country-wide campaign initiated by the government of India in 2014 [40]. The impact assessment study of India's Swachh Bharat Mission on acute diarrheal disease outbreaks, analysed acute diarrheal diseases from 2010 to 2018 and reported a decline in the pattern of acute diarrheal disease outbreaks during the intervention period between 2014-2018 [41, 42]. Increased numbers of illness episodes were noted in the monsoon and post-monsoon seasons (June–December), varying slightly from the results of other studies. This may be attributable to different climate patterns among regions and varied season definitions over time for a region [27, 43].

405 The present study also examined the effect of socio-demographic factors on the HSB of caregivers. The results from our study show that socio-demographic factors, such as the mother's education, occupation and socio-economic status were associated with where the caregivers sought healthcare and similar findings have also been reported in other studies [34, 44]. Maternal education levels and occupations are significant HSB determinants [34]. This may be due to the fact that educated mothers are more likely to have a better understanding of healthcare and

For peer review only - http://bmjopen.bmj.com/site/about/guidelines.xhtml

411 provide preventive and curative services for the child. A mother's education is associated with WASH (water, sanitation and hygiene) and with HSBs relevant to decreasing childhood mortality [45, 46].

414 Overall, the antibiotic prescription rate found in our study was 46%. Fewer antibiotics were prescribed in this study than has been reported in other studies [8-10]. This may be due to the fact that the majority of the illnesses reported in this study were RTIs where no treatment was given to the child in 44% of the episodes and home care was provided in 13%, suggesting that people in rural areas have some understanding about illness severity. Nevertheless, in our study results, 24% of the RTIs were cases of simple colds and coughs where antibiotics were prescribed, out of which 89% of the prescriptions were from IHCPs [5]. This implies inappropriate antibiotic prescribing according to the Indian National Treatment Guidelines for Antimicrobial Use in Infectious Diseases, which recommends antibiotics only for the treatment of pneumonia and tonsillitis, classified as RTIs, and for dysentery, classified under GI infections. Also 42% (n=335/807) of the total antibiotic course prescribied were of the "WATCH group antibiotics" classified under WHO AWaRe classification of antibiotics 2019. The classification was to emphasize the importance of optimal uses of antimicrobials and potential for antimicrobial resistance. WATCH group antibiotics includes antibiotics that have higher resistance potential and includes most of the highest priority agents among the Critically Important Antimicrobials for Human Medicine and/or antibiotics [47]. It has been stated that the healthcare in rural areas in India is predominantly served by informal healthcare providers, who have been reported as often prescribing antibiotics for common illnesess inappropriately [8, 29].

432 The longitudinal follow-up of caregiver HSB in the same cohort helped us to generate a detailed picture of healthcare-seeking pathways. This allowed us to understand several aspects of these

pathways, including delays providing treatment, variabilities in the caregiver pathways and factors affecting healthcare-seeking pathway. Repeated recordings of the HSB occasions from the same group of caregivers also increased reliability. In addition, repeated weekly data collection from the same group of caregivers could control the factors that cause variabilities between responses. Furthermore, the recall period of 2 days reduced the chances of recall bias. Information on the episodes of illness was provided by the mothers and thus was subjective in the sense that none of the episodes of illness were validated externally by any medical examination. But the classification of the sickness was performed by the trained research assistants, as evidence suggests that caregivers may be unable to accurately classify illnesses [48]. The quality of data is good and reliable. Providers co-operated in providing the details of the medicines depensed loose as they happen to remember the medicine whether or not they keep any resords for the same, still some of the medicines were not identifiable.

Conclusions

In our rural cohort for many acute episodes of illnesses no treatment or home treatment was done, which resulted in overall reduced antibiotic prescribing. The commonest pathway taken for seeking healthcare was visiting IHCPs which is suggestive of the fact that they are major healthcare providers in rural areas. Most of the antibiotics were prescribed by IHCPs, and were commonly prescribed for illnesses where they were not indicated.

Recommendations

Effective awareness and health education programmes at community level should be introduced to cater to illiterate and low-income populations to improve their healthcare-seeking practices. IHCPs should be trained in order to integrate them into the legal healthcare system, thus

For peer review only - http://bmjopen.bmj.com/site/about/guidelines.xhtml

strengthening the healthcare system, especially in rural areas. This can be done under the Prime Minister skill development program [49]. A pilot program in West Bengal has shown encouragingresults [39].

ETHICS STATEMENT

Patient consent for publication

Not required.

Ethical approval

The study was approved by the Institutional Ethics Committee of the RDGMC, Ujjain, India (No: 2013/07/17-311).

ACKNOWLEDGEMENT

The authors thank the participating caregivers for providing their valuable time. We would also like to thank Medical Director, Dr.V.K. Mahadikand the management of R.D. Gardi Medical College, Ujjain for providing administrative assistance for the study. We are also thankful to reseaech assistants Sunita, Vikram, Bharat, Pooja, Maya, Mamta, Mukesh, Bhaarat for assisting in data collection, project staff PriyankSoni, DevisSaha, VivekParashar for processing data and Ankit Garg and Giriraj Singh Sisodiya for database management.

DECLARATION OF INTEREST

None declared

AUTHOR CONTRIBUTIONS

VD together with MP, AP, MS, SK, AJT and CSL initiated the concept and formulated the initial design of the study. SK and VD are the main persons responsible for the study coordination. MP and MS provided the critical comments on the concept and design. SK and VD were responsible for data collection. SK performed data analysis. AP and GM supported the data analysis. SK drafted the first version of the manuscriptand constructed all tables and designed all figures. AP and VD commented on the first draft. AJT was the senior advisor to the project. CSL is the principal investigator for the project. All authors have commented earlier versions of the manuscript and read and approved the final version of the manuscript.

FUNDING

The project was funded by the Swedish Research Council (grant no 521-2012-2889). The funders had no role in study design, data collection and analysis, decision to publish, or preparation of the manuscript.

DATA AVAILABILITY

The datasets used and/or analysed during the current study are available from the corresponding author on reasonable request. Due to ethical and legal restrictions, all inquiries should be made with The Chairman, Ethics Committee, R.D. Gardi Medical College, Agar Road, Ujjain, India 456006 (Emails: iecrdgmc@yahoo.in, uctharc@bsnl.in), giving all details of the publication. For reference, please quote ethical permission no. 311, dated July 17, 2013.

FIGURES

Figure 1: Map showing geographical location of the study villages, formal/informal healthcare services within the 10 Km distance from the central village in Ujjain district, MP, India.

For peer review only - http://bmjopen.bmj.com/site/about/guidelines.xhtml

Figure 2: Distribution of total number of the episodes of illness recorded over the period of 113 weeks (with corresponding seasons)

Figure 3: Healthcare-seeking pathway of the caregivers of under-5 children, under study in rural Ujjain, India.

REFERENCES

1. **World Health Organization. Pre-Antibiotic era looming large - The world is almost out of time. 2014. http://www.searo.who.int/mediacentre/features/2014/rd-statement-amr2014/en/. Accessed on 1 Apr 2021**.

2. Levy SB, Marshall B: **Antibacterial resistance worldwide: causes, challenges and responses**. *Nat Med* 2004, **10**(12 Suppl):S122-129.

3. Meek RW, Vyas H, Piddock LJW: **Nonmedical Uses of Antibiotics: Time to Restrict Their Use?** *PLoS Biol* 2015, **13**(10):e1002266-e1002266.

4. **Child Global Burden of Disease, Adolescent Health, Reiner RC,Jr., Olsen HE, Ikeda CT, Echko MM, Ballestreros KE, Manguerra H, Martopullo I, Millear A, et al: Diseases, Injuries, and Risk Factors in Child and Adolescent Health, 1990 to 2017: Findings From the Global Burden of Diseases, Injuries, and Risk Factors 2017 Study**. *JAMA pediatrics* 2019, **173**(6):e190337.

5. **National treatment guidelines for antimicrobial use in infectious diseases. National center for disease control , Directorate General of Health Services, Ministry of**

Health & Family Welfare. Government of India 2016. http://pbhealth.gov.in/AMR_guideline7001495889.pdf. Accessed on 2 Dec 2020.

6. Pathak A, Upadhayay R, Mathur A, Rathi S, Lundborg CS: **Incidence, clinical profile, and risk factors for serious bacterial infections in children hospitalized with fever in Ujjain, India**. *BMC infectious diseases* 2020, **20**(1):162.

7. Pathak D, Pathak A, Marrone G, et al: **Adherence to treatment guidelines for acute diarrhoea in children up to 12 years in Ujjain, India--a cross-sectional prescription analysis**. *BMC infectious diseases* 2011, **11**:32.

8. Khare S, Purohit M, Sharma M, Tamhankar AJ, Lundborg CS, Diwan V, Pathak A: **Antibiotic Prescribing by Informal Healthcare Providers for Common Illnesses: A Repeated Cross-Sectional Study in Rural India**. *Antibiotics (Basel, Switzerland)* 2019, **8**(3).

9. Pathak A, Mahadik K, Dhaneria SP, et al: **Antibiotic prescribing in outpatients: Hospital and seasonal variations in Ujjain, India**. *Scandinavian journal of infectious diseases* 2011, **43**(6-7):479-488.

10. Sharma M, Damlin A, Pathak A, et al: **Antibiotic Prescribing among Pediatric Inpatients with Potential Infections in Two Private Sector Hospitals in Central India**. *PloS one* 2015, **10**(11):e0142317.

11. Costelloe C, Metcalfe C, Lovering A, et al: **Effect of antibiotic prescribing in primary care on antimicrobial resistance in individual patients: systematic review and meta-analysis**. *BMJ (Clinical research ed)* 2010, **340**:c2096.

For peer review only - http://bmjopen.bmj.com/site/about/guidelines.xhtml

12. Miller R, Goodman C: **Performance of retail pharmacies in low- and middle-income Asian settings: a systematic review**. *Health policy and planning* 2016, **31**(7):940-953.

13. Rao M, Rao KD, Kumar AK, et al: **Human resources for health in India**. *Lancet* 2011, **377**(9765):587-598.

14. **World Health Organization. Improving the containment of antimicrobial resistance. WHA. https://www.searo.who.int/entity/medicines/topics/wha_58_27.pdf. 2005. Accessed on 1 Apr 2021**.

15. Dekker AR, Verheij TJ, van der Velden AW: **Inappropriate antibiotic prescription for respiratory tract indications: most prominent in adult patients**. *Family practice* 2015, **32**(4):401-407.

16. Viberg N, Kalala W, Mujinja P, Tomson G, Lundborg CS: **"Practical knowledge" and perceptions of antibiotics and antibiotic resistance among drugsellers in Tanzanian private drugstores**. *BMC infectious diseases* 2010, **10**.

17. Conner M, Norman P: **The theory of planned behaviour and health behaviours. Predicting Health Behaviours: Research and Practice with Social Cognition Models. Buckingham: Open University Press**. 1996:121-162.

18. **Registrar General of India (2012) Census of India 2011. Government of India, New Delhi; http://www.censusindia.gov.in/vital_statistics/SRS_Reports_2012.html**.

19. Barik D, Thorat A: **Issues of Unequal Access to Public Health in India**. *Front Public Health* 2015, **3**:245-245.

For peer review only - http://bmjopen.bmj.com/site/about/guidelines.xhtml

20. Wattal C, Goel N: **Tackling antibiotic resistance in India**. *Expert review of anti-infective therapy* 2014, **12**(12):1427-1440.

21. **Encyclopedia Britannica. Madhya Pradesh [Internet]. Britannica Academic. https://academic.eb.com/levels/collegiate/article/MAdhya-Pradesh/111205 (2017). Accessed on 1 Apr 2021**.

22. **National Family Health Survey 4. 2015 -16. State Fact Sheet Madhya Pradesh. http://rchiips.org/NFHS/pdf/NFHS4/MP_FactSheet.pdf. Accessed on 1 Apr 2021**.

23. **Subnational Human Development Index - Area Database - Global Data Lab. Institute for Management Research Radbound University. 2018. https://www.https://globaldatalab.org/shdi/shdi/. Accessed on 1 Apr 2021.**

24. Stalsby Lundborg C, Diwan V, Pathak A, et al: **Protocol: a 'One health' two year follow-up, mixed methods study on antibiotic resistance, focusing children under 5 and their environment in rural India**. *BMC Public Health* 2015, **15**:1321.

25. Rowe AK, Lama M, Onikpo F, et.al.: **Design effects and intraclass correlation coefficients from a health facility cluster survey in Benin**. *International journal for quality in health care : journal of the International Society for Quality in Health Care* 2002, **14**(6):521-523.

26. Vyas S, Kumaranayake L: **Constructing socio-economic status indices: how to use principal components analysis**. *Health policy and planning* 2006, **21**(6):459-468.

For peer review only - http://bmjopen.bmj.com/site/about/guidelines.xhtml

27. Arora SK, Jha B: **Indian Meteorological Department 2018.** http://mausam.imd.gov.in/. **Accessed on 1 Apr 2021**.

28. **World Health Organization. Classifying health workers: Mapping occupations to the international standard classification.** https://www.who.int/hrh/statistics/Health_workers_classification.pdf. **2008. Accessed on 1 Apr 2021.**

29. Sudhinaraset M, Ingram M, Lofthouse HK, et al: **What is the role of informal healthcare providers in developing countries? A systematic review**. *PloS one* 2013, **8**(2):e54978.

30. WHO: **World Health Organization Anatomical Therapeutic Chemical (ATC) classification system: guidelines for ATC classification and DDD assignment 2018. WHO Collaborating Centre for Drug Statistics Methodology, ATC classification index with DDDs, 2018. Oslo, Norway**. 2018.

31. Balarajan Y, Selvaraj S, Subramanian SV: **Health care and equity in India**. *Lancet* 2011, **377**(9764):505-515.

32. Chauhan RC, Manikandan., Purty AJ, et al: **Determinants of health care seeking behavior among rural population of a coastal area in South India**. *Int J Scientific Reports* 2015, **1**(2):118-122.

33. Kalita B, Borah MK, Kakati R, et al: **Primary caregivers health seeking behaviour for under-five children: A study in a rural block of Assam, India**. *Ntl J Community Med* 2016.

For peer review only - http://bmjopen.bmj.com/site/about/guidelines.xhtml

34. Kanungo S, Bhowmik K, Mahapatra T, et al: **Perceived morbidity, healthcare-seeking behavior and their determinants in a poor-resource setting: observation from India**. *PloS one* 2015, **10**(5):e0125865.

35. Mackintosh M, Channon A, Karan A, Selvaraj S, Cavagnero E, Zhao H: **What is the private sector? Understanding private provision in the health systems of low-income and middle-income countries**. *Lancet* 2016, **388**(10044):596-605.

36. Peters DH, Rao KS, Fryatt R: **Lumping and splitting: the health policy agenda in India**. *Health policy and planning* 2003, **18**(3):249-260.

37. Satpathy SK: **Public health infrastructure in rural India: challenges and opportunities**. *Indian journal of public health* 2005, **49**(2):57-62.

38. Chandra S, Bhattacharya S: **Unqualified Medical Practitioners: Their Illegal but Indispensable Role in Primary Healthcare. Economic and Political Weekly, 2019. Vol LIV No. 5 (February 2, 2019): p. 36-44**.

39. Das J, Chowdhury A, Hussam R, et.al.: **The impact of training informal health care providers in India: A randomized controlled trial**. *Science* 2016, **354**(6308):aaf7384.

40. **Swachh Bharat Mission - Gramin, Department of Drinking water & Sanitation, Ministry of Jal Shakti, Government of India. https://www.swachhbharatmission.gov.in/SBMCMS/study-survey.htm. Accessed on 1 Apr 2021**.

41. **Review of health data in selected ODF and non-ODF districts under the SBM (Report of Findings). Ministry of Drinking Water and Sanitation (MoDWS), Government of India. 2017.** https://www.https://jalshakti-ddws.gov.in/sites/default/files/BMGF_Health_Impact_Study_final.pdf**. Accessed on 1 Apr 2021**.

42. Dandabathula G, Bhardwaj P, Burra M, Rao PVVP, Rao SS: **Impact assessment of India's Swachh Bharat Mission - Clean India Campaign on acute diarrheal disease outbreaks: Yes, there is a positive change**. *Journal of family medicine and primary care* 2019, **8**(3):1202-1208.

43. Reddaih VP, Kapoor SK: **Epidemiology of pneumonia in rural underfives**. *Ind J Com Medicine* 1990, **57**:701-704.

44. Chandwani H, Pandor J: **Healthcare-seeking behaviour of mothers regarding their children in a tribal community of Gujarat, India**. *Electronic Physician* 2015, **7**(1):990-997.

45. **Rebecca YS. DHS Comparative reports: child morbidity and treatment patterns. Calverton, Maryland: ORC macro, Calvetron, USA, 2004.** .

46. **Inter-Agency and Expert Group on MDG indicators. The millennium development goals report 2012. New York: United Nations, 2012.**

47. **World Health Organisation. WHO releases the 2019 AWaRe Classification Antibiotics. 2019.**

For peer review only - http://bmjopen.bmj.com/site/about/guidelines.xhtml

https://www.who.int/medicines/news/2019/WHO_releases2019AWaRe_classification_antibiotics/en/. **Accessed on 1 Apr 2021.**

48. Hazir T, Begum K, El Arifeen S, Khan AM, Huque MH, Kazmi N, Roy S, Abbasi S, Rahman QS, Theodoratou E *et al*: **Measuring coverage in MNCH: a prospective validation study in Pakistan and Bangladesh on measuring correct treatment of childhood pneumonia**. *PLoS medicine* 2013, **10**(5):e1001422.

49. India Go: **Schemes & Initiatives through NSDC. Pradhan Mantri Kaushal Vikas Yojana 2016-2020. Ministry of Skill Development and Entrepreneurship.** https://www.msde.gov.in/schemes-initiatives/schemes-initiatives-through-nsdc#:~:text=The%20PMKK%20program%20provides%20financial,Government%20at%20the%20district%20level. **Accessed on 1 Apr 2021**.

Table 1: Incidence and type of illness episodes; antibiotic prescribing and the first point of care recorded during healthcare-seeking behaviour follow up for the under-5 children in the study, in rural Ujjain

Variables	Total episodes N=2161	Healthcare-seeking behaviour				Illness episodes with antibiotics prescribed N=670
		No treatment N=711	Home care(a) N=316	Formal Healthcare(b) N=72	Informal Healthcare(c) N=1062	
**Illnesses reported**						
RTI^	1501	654 (44)	202 (13)	38 (3)	607 (40)	354 (24)
GIinfections^	182	8 (5)	31 (17)	17 (9)	126 (69)	82 (45)
Fever	147	4 (3)	28 (19)	4 (3)	111 (75)	52 (35)
Skin infections	138	14 (10)	12 (9)	7 (5)	105 (76)	86 (62)
Others	193	31 (16)	43 (22)	6 (3)	113 (59)	96 (50)
**Reasons for not taking treatment**						
Thought as illness is self-curable	-	333 (47)	-	-	-	-
Illness is mild	-	274 (39)	-	-	-	-
Others**	-	104 (14)	-	-	-	-
**Delay in seeking treatment (in days)**		-	**N=1450 (%)**			-
On the same day		-		1045 (72)		-
After one day		-		305 (21)		-
After two days		-		61 (4)		-
After three days		-		39 (3)		-

[a]Episodes of illness for which caregivers did not consult any healthcare provider and gave child home treatment (home remedies) or left over medicines or brought medicines from local shop on their own; [b] Episodes of illness for which caregiver consulted formal government/private practitioner, Government/private healthcare facility, community health visitor, Lady health visitor (ASHA/Anganwadi worker), Health camps; [c] Episodes of illness for which caregiver consulted informal private practitioner, local drug vendors without license, traditional healers; ^CI- Confidence Interval;SD- Standard Deviation; RTI- Respiratory Tract Infections; GI infections- Gastrointestinal infections

For peer review only - http://bmjopen.bmj.com/site/about/guidelines.xhtml

Table 2: Socio-demographic characteristics of mothers (caregivers) and under-5 children under study in rural Ujjain, India

Variables	Frequency (n)	Percentage (%)
**Child's Age (in years)(N=270)**		
≤ 1	140	52
2	61	23
3	23	8
4	26	10
5	20	7
**Child's Gender(N=270)**		
Male	136	50.4
Female	134	49.6
**Mother's Age (in years)(N=141)**		
14-25	88	62
> 25	53	38
**Education of mother(N=141)**		
Illiterate	71	50.4
School education and above*	70	49.6
**Occupation of mother(N=141)**		
Home maker	101	71.6
Working**	40	28.4
**Family members/ household(N=118)**		
≤ 5	45	38
> 5	73	62
**Parity (number of children)(N=141)**		
1-2	95	67
≥ 3	46	33
**Socio-economic status (PCA)(N=118)**		
Fifth Quintile (Wealthiest)	17	14
Fourth quintile (Wealthier)	23	19
Third quintile (Middle)	28	24
Second quintile (Poorer)	21	18
Fist quintile (Poorest)	29	25

^PCA- Principle component analysis; *School education includes primary school education from class 1 to class 8 and secondary school education from class 9 to class 12; **Working includes Farmer, Labour worker, Job in public/private sector

Table 3: Mixed-effects multinomial logistic regression model for predictors of healthcare-seeking behaviour of caregivers for common illnesses reported in under-5 children in study in Ujjain, India

Base outcome: "Formal healthcare" for the illness reported during healthcare-seeking behaviour follow-up

Variable	No treatment given (n=711)		Home treatment (n=316)		Informal healthcare (n=1062)	
	aRR^ (95% CI)^	p value	aRR^ (95% CI)^	p value	aRR^ (95% CI)^	p value
**Illness Type**						
Other illness	1	-	1	-	1	-
RTI#	11.54 (6.14-21.71)	<0.001*	1.82 (1.03-3.24)	0.04*	1.29 (0.75-2.23)	0.36
**Education of mother**						
School education and above‡	1	-	1	-	1	-
Illiterate	2.86 (1.41-5.80)	0.004*	2.38 (1.21-4.68)	0.01*	1.93 (0.90-3.74)	0.05
**Occupation of mother**						
Working**	1	-	1	-	1	-
Home maker	2.90 (1.43-5.90)	0.004*	4.17 (2.06-8.46)	<0.001*	2.10 (1.16-3.79)	0.02*
**Socio-economic status (PCA)**						
Fifth Quintile (Wealthiest)	1	-	1	-	1	-
Fourth quintile (Wealthier)	3.56 (1.30-9.77)	0.01*	3.24 (1.39-7.58)	0.007*	1.90 (0.81-4.45)	0.14
Third quintile (Middle)	3.51 (1.31-9.37)	0.01*	4.52 (1.72-11.87)	0.002*	3.20 (1.33-7.67)	0.01*
Second quintile (Poorer)	6.59 (2.15-20.23)	0.001*	9.20 (3.55-23.81)	<0.001*	4.38 (1.50-12.90)	0.01*
First quintile (Poorest)	6.39 (2.13-18.86)	0.001*	6.86 (2.62-18.01)	<0.001*	3.19 (1.23-8.26)	0.02*

^aRR: Adjusted Risk ratios, CI: Confidence Interval; *Significant p value <0.05; ‡ School education includes primary school education from class 1 to class 8 and secondary school education from class 9 to class 12; **Working includes Farmer, Labour worker, Job in public/private sector; n = number of yes response; #RTI: acute respiratory tract infection

For peer review only - http://bmjopen.bmj.com/site/about/guidelines.xhtml

Table 4: Number of illness episodes with antibiotic prescriptions categorised using anatomical therapeutic chemical (ATC) classification, reported during the healthcare-seeking behaviour follow up in study in Ujjain, India

Illnesses	Penicillin with extended spectrum; amoxicillin and ampicillin with cloxacillin J01CA n= 94(%)	Penicillin with β -lactamase inhibitor, amoxicillin with clavulanic acid J01CR n= 59	Tetracyclines J01A n= 2	Cephalosporins; J01D 1st generation J01DB n= 27	Cephalosporins; J01D 2nd generation J01DC n= 4	Cephalosporins; J01D 3rd generation J01DD n= 259	Sulfonamides and Trimethoprim J01E n= 50	Aminoglycosides J01GB n= 3	Macrolides J01FA n= 19	Fluoroquinolones J01MA n= 86	Metronidazole Nitazoxanide (P01AX11)P01 AB n= 12	Combinations of antibacterial J01RA n= 39
RTI* (n=1501)	59(61)	33(56)	1(50)	18(67)	2(50)	173(67)	23(46)	1(67)	10(52)	33(38)	4(33)	18(46)
GI infections** (n=182)	8(9)	10(17)	-	3(11)	-	21(8)	1(2)	-	2(11)	15(18)	8(67)	15(38)
Fever (n=147)	9(10)	9(15)	-	3(11)	1(25)	23(9)	3(6)	-	3(16)	6(7)	-	2(5)
Skin infections (n=138)	10(10)	4(7)	1(50)	2(7)	-	15(6)	17(34)	-	3(16)	20(23)	-	3(8)
Others (n=193)	8(9)	3(5)	-	1(4)	1(25)	27(10)	6(12)	2(33)	1(5)	12(14)	-	1(3)

*** RTI- Respiratory tract infections, **GI infections- gastrointestinal infections; n- frequency of the respective category; Column percentages are corresponding to the column total; Row percentage will not match as more than one antibiotics were prescribed for the respective illness types in some prescriptions**

List of abbreviations

RTI - Acute respiratory tract infections

HSB - Healthcare-seeking behaviour

IHCPs - Informal healthcare providers

U-5 - Under-5

MP - Madhya Pradesh

DSS - Demographic surveillance site

RDGMC –RuxmanibenDeepchandGardi Medical College

GI - Gatro-intestinal

ATC - Anatomical Therapeutic Chemical Classification

aRR - Adjusted relative risk

CI - Confidence interval

SD - Standard deviation

aOR-Adjusted odds ratio

WHO – World Health Organisation

For peer review only - http://bmjopen.bmj.com/site/about/guidelines.xhtml

Figure 1: Map showing geographical location of the study villages, formal/informal healthcare services within the 10 Km distance from the central village in Ujjain district, MP, India.

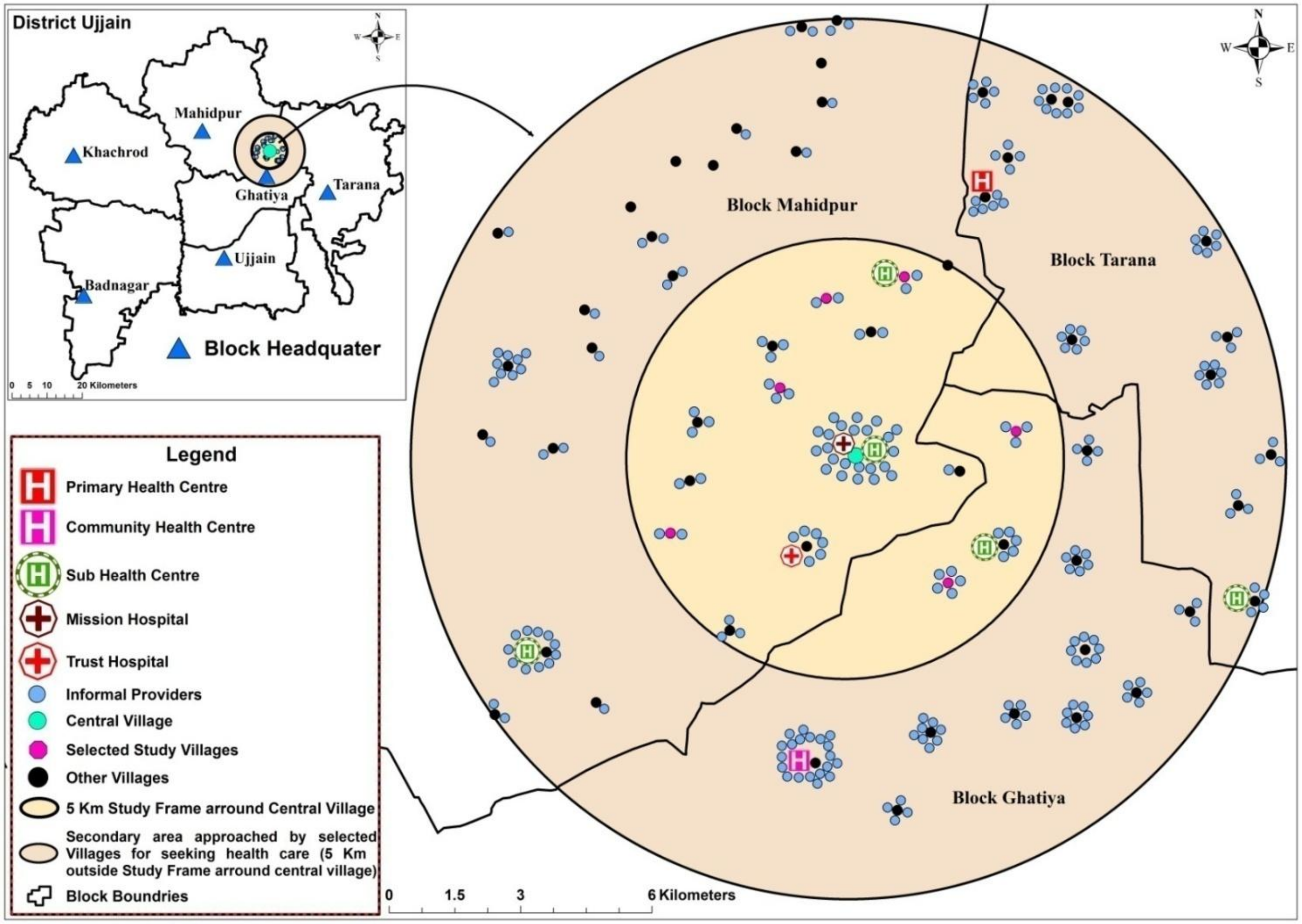

Map showing 5 Km study frame around the central village in Ujjain district, MP, India with the geographical location of the study villages, informal healthcare providers practising within the study frame and formal healthcare facilities located within the 10 Km distance from the central village within study frame in the inset map. (Source: Survey of India (SOI) in Dehra Dun, India (License No. BP11CDLA183)

Figure 2: Distribution of total number of the episodes of illness recorded over the period of 113 weeks (with corresponding seasons)

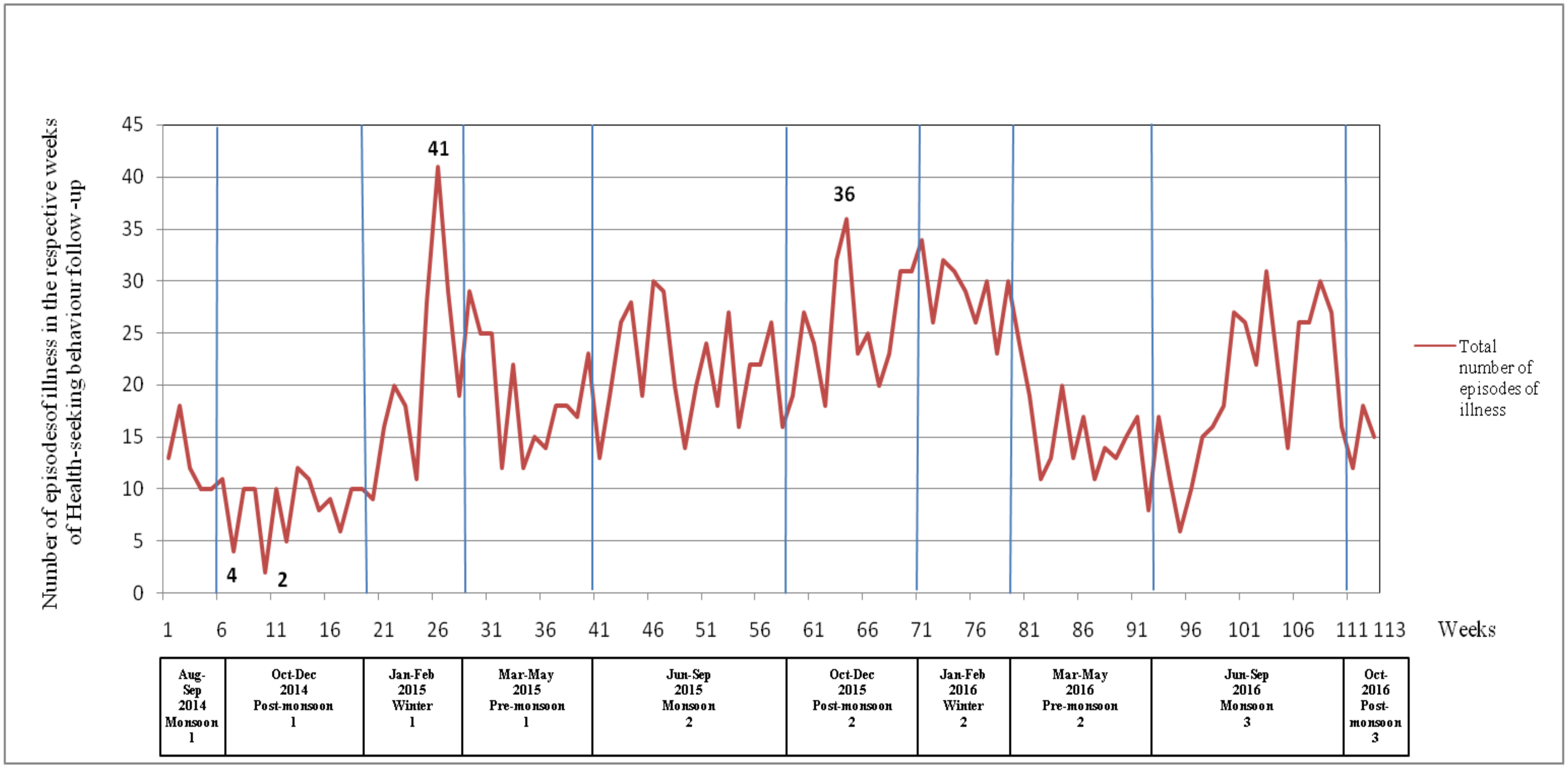

The X-axis represents the week from week 1 to week 113 (i.e, August 2014 to October 2016)with the corresponding seasons (August-September 2014 Monsoon 1, October-December 2014 Post-monsoon 1, January-February 2015 Winter 1, March-May 2015 Pre-monsoon 1, June-September 2015 Monsoon 2, October-December 2015 Post-monsoon 2, January-February 2016 Winter 2, March-May 2016 Pre-monsoon 2, June-September 2016 Monsoon 3, October 2016 Post-monsoon 3); Y-axis represents the number of episodes of illness reported during the healthcare-seeking behaviour follow-up in the respective week

Figure 3: Healthcare-seeking pathway of the caregivers of under-5 children, under study in rural Ujjain, India.

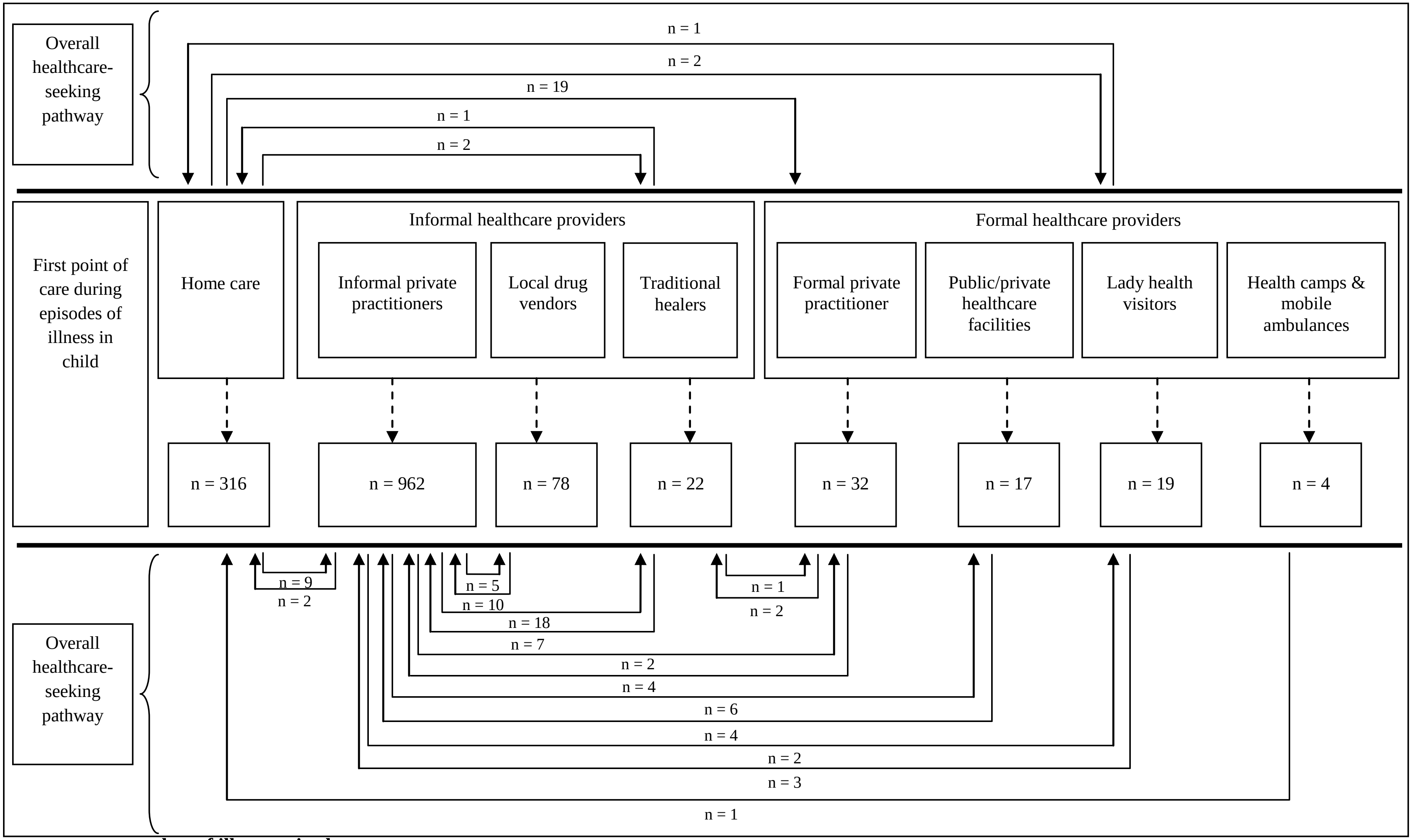

n= number of illness episodes

Additional File 1: Socio-demographic survey questionnaire

Part1: Information on household members:

S.No.	Name	Sex (M/F)	Age	Relation with 'Head'	Educational Qualification	Occupation
1.						
2.						
3.						
4.						
5.						
6.						
7.						
8.						
9.						
10.						

Part2: Questionnaire

Gender of head of household
Respondent age
Gender of respondent
Family type
Number of family members
Number of children of age 0 to 1 year, 1 to 3 years, 0 to 5 years, 6 to 15 years
Number of members above 15 years of age
Type of caste
Which ration card does the family have
Type of house (kuccha, semi-kuccha, pucca)?
Do you own your house?
Number of rooms other than kitchen, toilet, storage
Do you pay bill for electricity
First priority source of electricity used
Second priority source of electricity used
Third priority source of electricity used
Fourth priority source of electricity used
Does your household possess:
- Radio
- Television
- Bicycle
- Motor cycle
- Bull/horse
- Car/jeep
- Tractor
- Harvester
- Generator

For peer review only

LPG
Water pump in house
water pump in field
Landline (Phone)
Mobile (Phone)
Internet
Cooler, table fan or Ceiling fan
Fridge
Diesel Engine
Livestock
Do you own any property and agriculture land?
If yes - How much property other than agriculture in square feet (sq.ft)?
If yes - How much is for agriculture in sq.ft?
First priority main source of drinking water
What kind of toilet facility do members of your household usually use?

For peer review only

Additional File 2: Healthcare-seeking behavior diary

S.NO.	QUESTIONS
	**Section A: General information about illness**
1.	History of presenting illness
2.	Did you seek any treatment for your child or gave anything on your own to your child for the illness?
3.	If **'NO'**, Why? **(end the interview)**
4.	If **'YES'**, Where?
	**Section B: Treatment at "HOME"**
5.	If 'HOME', describe the treatment?<br>**(if 'NO' skip to 12)**
6.	What medicine you gave your child? mention the type, dose and source **(ask for**
7.	Who advice you the above mentioned treatment for your child's illness?
8.	From where did you get the medicine **(over the counter etc)?**
9.	Estimated direct cost of home treatment **(in rupees)**
10.	Did your child got relief from the illness **(YES / NO)**
11.	If **'NO'**, where did you took your child next for the treatment
12.	Name of '**Healthcare facility/private practitioner/CHV/ LHV/Pharmacist** etc.' visited for
13.	Where the healthcare facility/ clinic are located?
14.	Why you took your child to this healthcare facility/ private practitioner?
15.	Mode of transport to reach the facility?
16.	Travel time **(in minutes)**
17.	Did they prescribe any medicine?
18.	If **'YES'** describe the medicine with the respective dose /provide prescription?
19.	Did you give your child the complete dose of prescribed medicine? **(YES/NO)**<br>**(if 'YES' skip to 21)**
20.	Why you did not gave the prescribed dose to your child?
21	Were there any adverse events from any of the medicines prescribed?
22.	Did your child have any relief from the illness?<br>**(YES / NO)**
23.	Were your child asked for any investigation? **(YES/NO)**<br>AndIf**"YES"** mention the type of investigation?<br>**(if 'NO' skip to 25)**
24.	Did you take your child for the investigations? **(YES/NO)**
25.	Estimated direct cost of treatment from healthcare facility / private practitioner<br>**(excluding the travel cost) (in rupees)**
26.	Transport Cost to reach healthcare facility/ private practitioner
	**Section D: Treatment by "Traditional healers"**
27.	Did you seek any treatment from the Traditional healers for your child's illness? **(YES/NO)**
28.	What kind of treatment was provided by them? Describe treatment
29.	Did your child got any relief from the illness? **(YES/NO)**
30.	Where do you generally seek the treatment for your child when he/she gets common cold, cough, fever, body ache?

For peer review only

Additional File 3: Presenting illness classification

**Presenting illness classification**	**Key illnesses under presenting illness classification**
RTI*	Cold,cough with/without fever,ear infection, tonsillitis
GI infections*	Diarrhoea with/without stomach pain, appetite loss vomiting, gas, constipation
Fever	Fever without other illness
Others	Illnesses that occurred rarely e.g. chicken pox, ring worm, injury, insect/animal bite, oral problems

* RTI- Acute respiratory tract infections, GI infections- Gastrointestinal infections

For peer review only

Additional File 4: Flow chart describing sample population, episodes of illness recorded among study population, under study in rural Ujjain, India.

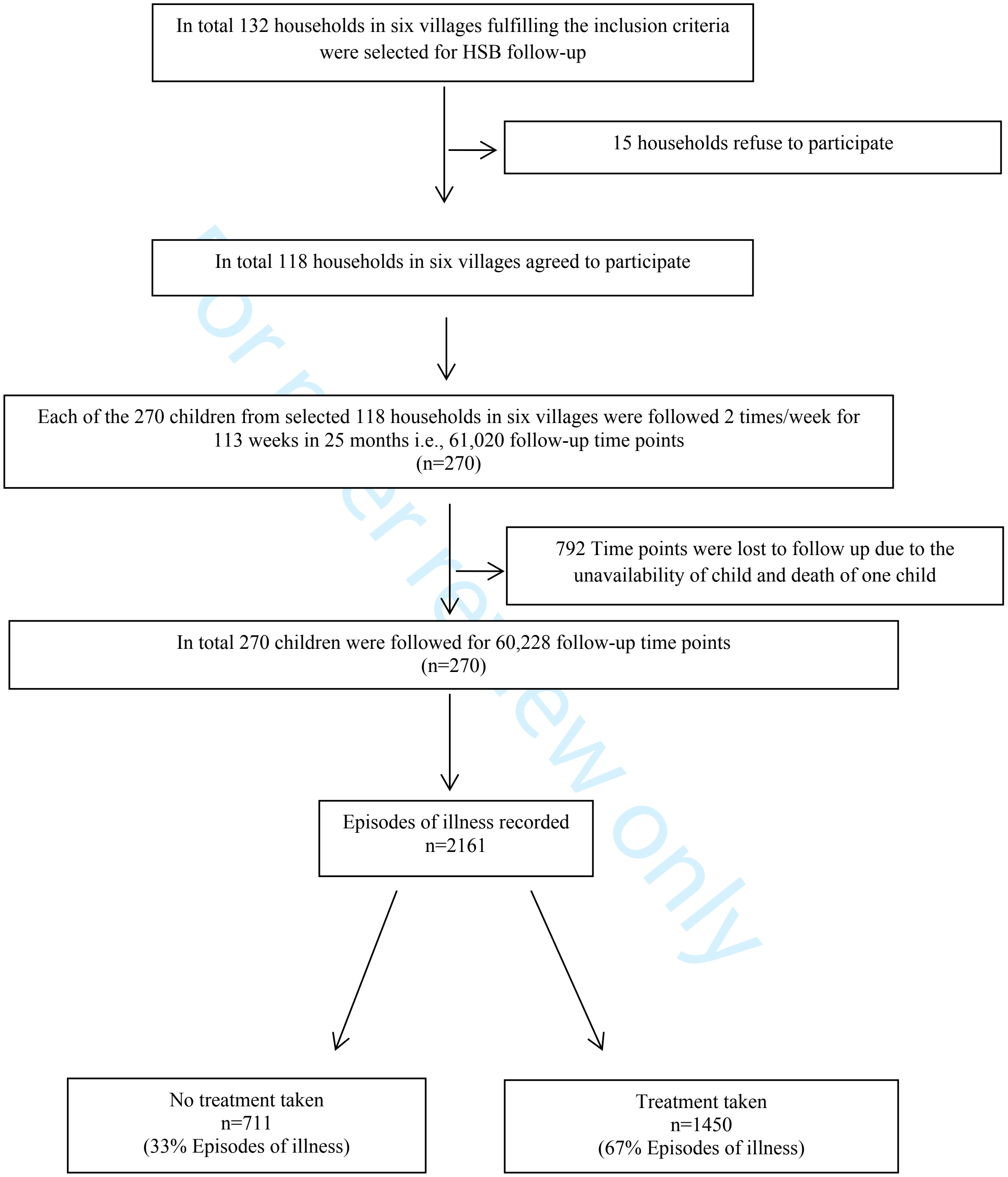

For peer review only

STROBE Statement—Checklist of items that should be included in reports of ***cohort studies***

	Item No	Recommendation	Page No
**Title and abstract**	1	(*a*) Indicate the study's design with a commonly used term in the title or the abstract	1
		(*b*) Provide in the abstract an informative and balanced summary of what was done and what was found	2-4
**Introduction**			
Background/rationale	2	Explain the scientific background and rationale for the investigation being reported	5-6
Objectives	3	State specific objectives, including any prespecified hypotheses	6
**Methods**			
Study design	4	Present key elements of study design early in the paper	7
Setting	5	Describe the setting, locations, and relevant dates, including periods of recruitment, exposure, follow-up, and data collection	7-10
Participants	6	(*a*) Give the eligibility criteria, and the sources and methods of selection of participants. Describe methods of follow-up	7-8
		(*b*)For matched studies, give matching criteria and number of exposed and unexposed	
Variables	7	Clearly define all outcomes, exposures, predictors, potential confounders, and effect modifiers. Give diagnostic criteria, if applicable	10-11
Data sources/ measurement	8*	For each variable of interest, give sources of data and details of methods of assessment (measurement). Describe comparability of assessment methods if there is more than one group	11-13
Bias	9	Describe any efforts to address potential sources of bias	20-21
Study size	10	Explain how the study size was arrived at	8
Quantitative variables	11	Explain how quantitative variables were handled in the analyses. If applicable, describe which groupings were chosen and why	10-13
Statistical methods	12	(*a*) Describe all statistical methods, including those used to control for confounding	13
		(*b*) Describe any methods used to examine subgroups and interactions	
		(*c*) Explain how missing data were addressed	
		(*d*) If applicable, explain how loss to follow-up was addressed	
		(*e*) Describe any sensitivity analyses	
**Results**			
Participants	13*	(a) Report numbers of individuals at each stage of study—eg numbers potentially eligible, examined for eligibility, confirmed eligible, included in the study, completing follow-up, and analysed	14
		(b) Give reasons for non-participation at each stage	14
		(c) Consider use of a flow diagram	14
Descriptive data	14*	(a) Give characteristics of study participants (eg demographic, clinical, social) and information on exposures and potential confounders	14
		(b) Indicate number of participants with missing data for each variable of interest	14
		(c) Summarise follow-up time (eg, average and total amount)	14
Outcome data	15*	Report numbers of outcome events or summary measures over time	15-16

For peer review only - http://bmjopen.bmj.com/site/about/guidelines.xhtml

Main results	16	(*a*) Give unadjusted estimates and, if applicable, confounder-adjusted estimates and their precision (eg, 95% confidence interval). Make clear which confounders were adjusted for and why they were included<br>(*b*) Report category boundaries when continuous variables were categorized<br>(*c*) If relevant, consider translating estimates of relative risk into absolute risk for a meaningful time period	17
Other analyses	17	Report other analyses done—eg analyses of subgroups and interactions, and sensitivity analyses	-
**Discussion**			
Key results	18	Summarise key results with reference to study objectives	18-20
Limitations	19	Discuss limitations of the study, taking into account sources of potential bias or imprecision. Discuss both direction and magnitude of any potential bias	20-21
Interpretation	20	Give a cautious overall interpretation of results considering objectives, limitations, multiplicity of analyses, results from similar studies, and other relevant evidence	21-22
Generalisability	21	Discuss the generalisability (external validity) of the study results	-
**Other information**			
Funding	22	Give the source of funding and the role of the funders for the present study and, if applicable, for the original study on which the present article is based	23

*Give information separately for exposed and unexposed groups.

Note: An Explanation and Elaboration article discusses each checklist item and gives methodological background and published examples of transparent reporting. The STROBE checklist is best used in conjunction with this article (freely available on the Web sites of PLoS Medicine at http://www.plosmedicine.org/, Annals of Internal Medicine at http://www.annals.org/, and Epidemiology at http://www.epidem.com/). Information on the STROBE Initiative is available at http://www.strobe-statement.org.

For peer review only

BMJ Open

Determinants and pathways of healthcare-seeking behaviours in under-5 children for common childhood illnesses and antibiotic prescribing: A cohort study in rural India

Journal:	*BMJ Open*
Manuscript ID	bmjopen-2021-052435.R1
Article Type:	Original research
Date Submitted by the Author:	23-Sep-2021
Complete List of Authors:	khare, shweta; Karolinska Institute, Department of Global Public Health ; Ruxmaniben Deepchand Gardi Medical College, Department of Public Health and Environment<br>Pathak, Ashish; Karolinska Institute, Department of Global Public Health ; Ruxmaniben Deepchand Gardi Medical College, Department of Pediatrics<br>Purohit, Manju; Karolinska Institute, Department of Global Public Health ; Ruxmaniben Deepchand Gardi Medical College, Department of Pathology<br>Sharma, Megha; Karolinska Institutet, Department of Global Public Health; RD Gardi Medical College, Department of Pharmacology<br>Marrone, Gaetano; Karolinska Institutet, Global Health (IHCAR)/Department of Global Public Health<br>Tamhankar, Ashok ; Karolinska Institute, Department of Global Public Health; Ruxmaniben Deepchand Gardi Medical College, Department of Environmental Medicine<br>Lundborg, Cecilia; Karolinska Institutet, Department of Global Public Health<br>Diwan, Vishal; Karolinska Institutet, Department of Global Public Health; ICMR- National Institute for Research in Environment Health
**Primary Subject Heading**:	Public health
Secondary Subject Heading:	Infectious diseases, Paediatrics
Keywords:	Quality in health care < HEALTH SERVICES ADMINISTRATION & MANAGEMENT, Public health < INFECTIOUS DISEASES, Community child health < PAEDIATRICS, PRIMARY CARE, PUBLIC HEALTH

SCHOLARONE™
Manuscripts

I, the Submitting Author has the right to grant and does grant on behalf of all authors of the Work (as defined in the below author licence), an exclusive licence and/or a non-exclusive licence for contributions from authors who are: i) UK Crown employees; ii) where BMJ has agreed a CC-BY licence shall apply, and/or iii) in accordance with the terms applicable for US Federal Government officers or employees acting as part of their official duties; on a worldwide, perpetual, irrevocable, royalty-free basis to BMJ Publishing Group Ltd ("BMJ") its licensees and where the relevant Journal is co-owned by BMJ to the co-owners of the Journal, to publish the Work in this journal and any other BMJ products and to exploit all rights, as set out in our licence.

The Submitting Author accepts and understands that any supply made under these terms is made by BMJ to the Submitting Author unless you are acting as an employee on behalf of your employer or a postgraduate student of an affiliated institution which is paying any applicable article publishing charge ("APC") for Open Access articles. Where the Submitting Author wishes to make the Work available on an Open Access basis (and intends to pay the relevant APC), the terms of reuse of such Open Access shall be governed by a Creative Commons licence – details of these licences and which Creative Commons licence will apply to this Work are set out in our licence referred to above.

Other than as permitted in any relevant BMJ Author's Self Archiving Policies, I confirm this Work has not been accepted for publication elsewhere, is not being considered for publication elsewhere and does not duplicate material already published. I confirm all authors consent to publication of this Work and authorise the granting of this licence.

For peer review only

Determinants and pathways of healthcare-seeking behaviours in under-5 children for common childhood illnesses and antibiotic prescribing: A cohort study in rural India

Shweta Khare[1,2#], Ashish Pathak[1,4,5], Manju Purohit[1,6], Megha Sharma[1,7], Gaetano Marrone[1], Ashok J Tamhankar[1,8], Cecilia Stålsby Lundborg[1], Vishal Diwan[1,3]

[1]Department of Global Public Health, Health Systems and Policy: Medicines focusing antibiotics, Karolinska Institutet, Tomtebodavagen18A, SE-171 77 Stockholm, Sweden

[2]Department of Public Health and Environment, RD Gardi Medical College, Ujjain, 456006, Madhya Pradesh, India

[3]ICMR- National Institute for Research in Environment Health, Bhopal, 462030, Madhya Pradesh, India

[4]Department of Pediatrics, RD Gardi Medical College, Ujjain, 456006, Madhya Pradesh, India

[5]Department of Women and Children's Health, International Maternal and Child Health Unit, Uppsala University, Uppsala SE-751 85, Sweden

[6]Department of Pathology, RD Gardi Medical College, Ujjain, 456006, Madhya Pradesh, India

[7]Department of Pharmacology, RD Gardi Medical College, Ujjain, 456006, Madhya Pradesh, India

[8]Indian Initiative for Management of Antibiotic Resistance, Department of Environmental Medicine, RD Gardi Medical College, Ujjain, 456006, Madhya Pradesh, India

[#]Corresponding author email: shweta.khare@ki.se; contact number: +91-9893986241

KEYWORDS: Healthcare-seeking behavior, Healthcare-seeking pathways, Common childhood illness, Carergivers', Under-5 children, Antibiotic prescribing, Rural population, India

WORD COUNT: 4956

REFERENCE COUNT: 49

ABSTRACT

Objectives

To explore the healthcare-seeking pathways, antibiotic prescribing and determine the socio-demographic factors associated with healthcare-seeking behaviour(HSB) of caregivers for common illnesses in under-5(U-5) children in rural Ujjain, India.

Study design Prospective cohort study.

Study setting and study sample

The cohort included 270 U-5 children from selected six villages in rural demographic surveillance site, of the R.D.Gardi Medical College, Ujjain, Madhya Pradesh, India. A community-based cohort was visited twice weekly for over 113 weeks(August 2014-October 2016) to record the HSB of caregivers using HSB diaries. Socio-demographic information was also solicited.

Primary and secondary outcome measures

Primary outcomes: First point of care, healthcare-seeking pathway and quantify antibiotic prescribing for the common acute illnesses.

Secondary outcome: HSB risk factors determined using mixed-effects multinomial logistic regression

Results

A total of 60,228 HSB follow-up time points for 270 children were recorded with a total of 2,161 acute illness episodes. The commonest illnesses found were RTI(69%)andGI(8%). No healthcare was sought in 33% of illness episodes, mostly for RTIs. The commonest healthcare-seeking pathway was to informal healthcare providers(IHCPs,49% of illness episodes). The adjusted relative risk(aRR) for obtaining no treatment, home treatment and treatment by IHCPs was higher for RTIs(aRR=11.54,1.82and1.29 respectively), illiterate mothers(aRR=2.86,2.38and1.93 respectively), and mothers who were homemakers(aRR=2.90,4.17and2.10 respectively). Socio-economic status was associated with HSB, with the highest aRR for no treatment in the lowest two socio-economic quintiles(aRR=6.59and6.39 respectively). Antibiotics were prescribed in 46%(n=670/1450) illness episodes and the majority (85%,n=572/670) were broad spectrum.

Conclusion

In our rural cohort for many acute episodes of illnesses no treatment or home treatment was done, which resulted in overall reduced antibiotic prescribing. The commonest healthcare-seeking pathway was to visit IHCPs, which indicates that they are major healthcare providers in rural areas. Most of the antibiotics were prescribed by IHCPs and were commonly prescribed for illnesses where they were not indicated.

For peer review only - http://bmjopen.bmj.com/site/about/guidelines.xhtml

ARTICLE SUMMARY

Strengths and limitations

- This is the largest study ever to prospectively follow the healthcare-seeking behaviour of caregivers of a cohort of U-5 children and antibiotic prescribing for common childhood illnesses, in a rural community setting in India.
- The longitudinal follow-up with repeated recordings of the same cohort's healthcare-seeking behaviour made the data reliable.
- Determined the pathway in healthcare-seeking behaviour for acute childhood illnesses, which have not been reported in studies from India.
- Information on the episodes of illnesses was subjective in nature and were not validated externally by any medical examination.
- The study did not evaluate the severity of the disease.

INTRODUCTION

Resistance to antibiotics has become a major threat to global health [1]. The crisis of antibiotic resistance compromises the ability to treat bacterial infections, which were once thought to have been contained [2]. Antibiotics are inappropriately used in humans, in animals as growth promoters, and also in aquaculture [3]. However, the greatest concern is for human use as this affects us directly.

Globally, 5.4 million children die before the age of 5 years per year, and about 50% of these deaths are caused by infectious diseases [4]. The commonest infections in children include acute respiratory tract infections (RTIs) and acute diarrhoea, both of which are more often a result of viral infections, for which antibiotic treatment is not recommended [5-7]. However, antibiotics are often prescribed incorrectly and for un-indicated conditions, for example in viral infections [8-10]. Higher rates of antibiotic prescribing are associated with higher rates of antibiotic resistance among the bacterial pathogens that cause common infections [11].

India has one of the largest three-tier healthcare systems (primary, secondary, tertiary) in the world. Each of the 3 levels of the healthcare system consist of both public and private healthcare providers which are a mix of allopathic and AYUSH (Ayurvedic, Unani, Siddha, Homeopathic - the non-allopathic systems of medicine practiced in India) practitioners. The practitioners of all the above systems are reported to be prescribing antibiotics. Antibiotics are also prescribed and/ or dispensed by the informal healthcare providers (IHCPs) and pharmacist [8, 12]. Antibiotics can also be purchased “over the counter” without prescription despite a law against it [8, 13]. Antibiotic prescribing rates in any community can be affected by the prescribing practices of healthcare providers, which are influenced by multiple factors [14-16] and the healthcare-seeking

behaviour (HSB) of the community. Studies have focused on how to influence the prescribing of antibiotics, but the factors affecting the HSB of a community have not been widely studied in regards to antibiotic prescribing. HSB includes any action or decision taken by a caregiver to regain the good health of a sick child [17]. In India, more than 60% of the population lives in rural areas [18], yet India's access to health care facilities is significantly urban biased [19]. Following the decision to seek healthcare, the subsequent pathway followed within the healthcare system often encompasses all available healthcare options, beginning with home care and traditional healers and extending to informal or formal health care services [20]. Gaining an understanding of the HSB of an individual is complex, and this behaviour is affected by multiple factors, including how medicine is practiced by existing healthcare providers, geographical location and socio-demographic factors [14-16]. These factors also influence antibiotic prescribing and are thus related to the development of antibiotic resistance [14-16].

Currently, there is limited literature on the determinants of HSB and antibiotic prescribing in rural areas, and furthermore, the pathway in HSB for acute childhood illnesses in India has not been studied. Therefore, the primary objective of the present prospective cohort study was to explore these healthcare-seeking pathways, investigate the HSB of caregivers and antibiotic prescribing for common acute infectious illnesses in under-5 (U-5) children. The secondary objective was to determine the socio-demographic factors associated with caregiver HSB following healthcare in the rural areas of Ujjain, India.

METHODS

Study design

Prospective cohort study with repeated follow-ups performed for 113 weeks from August 2014 to October 2016.

Study setting

This study was conducted in rural areas of the Ujjain district of Madhya Pradesh (MP) in central India. MP is India's second largest state by area and has 72 million inhabitants, with about 72% of the population residing in rural areas [21]. MP has poor health indicators (i.e., infant mortality rate of 51/1,000 live births, U-5 mortality rate of 65/1,000 live births [22]) and a human development index of 0.606 [23]. In this study, the rural demographic surveillance site (DSS) Palwa of Ruxmaniben Deepchand Gardi Medical College (RDGMC) was used. Six out of 60 villages were selected from this DSS, one of which was assigned as a central village. The six villages were selected using the following inclusion criteria: a) aerial distance of less than 5km from the central village, b) a population of at least 500 inhabitants c) and at least 15 children available in the desired age range of ≤5 years [24]. The map in Figure 1 shows the 5km study frame around the central village along with geographical location of the study villages, practising IHCPs and formal healthcare facilities. In addition, IHCPs and formal healthcare facilities at a 5km distance outside the study frame are also shown. Further details of the study settings are described elsewhere [24]. In the study formal healthcare providers/practitioners are the one including medical practitioners recognized by the Indian Medical Council working in a government or private healthcare facility and government certified healthcare workers including community health visitors, lady health visitors [25], whereas, IHCPs are the healthcare providers who have not received a formal degree in medicine from any institution and who are not registered as healthcare practitioners by any governing body, these includes informal private practitioners, local unlicensed pharmaceutical vendors and traditional healers [26].

Study cohort

After selecting the 6 villages as described above all the households were listed. A total 132 households in the six villages satisfied the inclusion criteria: a) had children U-5 and of having lived in the villages for the past one year, b) planned to live in the same village for the next two years and c) had parents consenting to participate in the study. Therefore, total of 118 households having 270 U-5 children were included in the study. All children from a given family were included if eligible.

Sample size calculation

Estimates of proportions from a single batch of 100 observations (e.g., of children) have a precision of +/-10%. Using repeated batches of 100 children per batch (each week per month) has a power of 80% for detecting a linear trend for any observation in proportion of 0.1 per time unit (step between batches, negative or positive). A conservative estimate of a design effect of 2 was deemed appropriate in consideration of the differences in population sizes among the selected villages [7, 27], giving a required minimum sample size of (100×2) 200 children. The number of children included in the cohort was increased by 10% to accommodate for possible attrition over time. Thus, a minimum sample size of 220 children could provide a sufficiently high precision in the estimates and a power of at least 80% to the comparisons between data points.

Data collection

Healthcare-seeking behaviour follow-up

169 At baseline, the socio-demographic characteristics of all the selected families were collected through a questionnaire that surveyed household amenities and assets and family member education levels and occupations **(Additional File 1)**. HSB survey forms, called 'healthcare-seeking behaviour diaries', were designed to meet the study requirements and piloted and checked for face validity **(Additional File 2)**.

Furthermore, a pilot study was done to test the data collection instrument and associated procedures and to train and prepare the research assistants. No changes were deemed necessary in the data collection instrument after the pilot. In the main study, the HSBs of caregivers for acutely sick children were recorded twice a week by trained research assistants familiar with the local language. An acutely sick child was defined according to the perceptions of the caregiver. The classification of the sickness was performed by the research assistants and not by the caregiver. The follow-ups were completed using HSB diaries by the research assistants for 113 consecutive weeks between August 2014 and October 2016. An entry in the HSB diary was created for the duration of an illness irrespective of the fact that the illness may have started prior to the research assistant's last visit. One HSB diary was maintained per child, so if a household had more than one participating child, a corresponding number of diaries were maintained. The entries in the HSB diary included the relevant history related to an episode of illness, whether or not any home treatment or clinic or hospital-based treatment was provided, the child's route through different healthcare systems following an illness episode, the reasons for taking those routes when seeking healthcare, treatment details with a focus on any prescribed antibiotics and treatment costs and hospitalisations. The informal healthcare providers usually dispense medicines to the patient by themselves and so do not provide any written prescriptions. In cases where prescriptions were not available the caregivers were asked to retain the labelled

For peer review only - http://bmjopen.bmj.com/site/about/guidelines.xhtml

wrappers/bottles of the medicines prescribed by the treating healthcare provider, and for the medicines which were dispensed loose, the details of such medicines and injections were asked from IHCPs. The entries in the HSB diaries were updated on subsequent biweekly visits until the episode of illness was resolved/cured or the caregiver stopped providing any treatment for that episode. Thus, one entry in the HSB diary was kept for each episode of illness per child. A new entry in the HSB diary was made for new signs and symptoms or if old signs and symptoms developed again after one day of recovery. Diaries were checked and completed for missing information for an episode of illness twice weekly by the first author.

Data management

Independent variables

The independent variables for the study were: a) illnesses recorded during the HSB follow-up, classified as RTIs, gastrointestinal (GI) infections, fever, skin infections and others (details are presented in **Additional File 3**), b) age of child in years, c) age of caregiver in years as ≤25 years versus >25 years, d) education of mother as illiterate versus school education and higher (where school education included primary school education from class 1 to class 8 and secondary school education from class 9 to class 12); e) occupation of mother as a home maker versus working (including farmers, labourers, and public/private sector jobs); f) number of family members as ≤5 members versus >5 members, g) parity of mother categorised as 1 child, 2 children and ≥3 children and h) socio-economic status which was determined using the principal component analysis approach as referenced [28] and was categorized as: first quintile (poorest), second quintile (poorer), third quintile (middle), fourth quintile (wealthier), and fifth quintile (wealthiest).

For peer review only - http://bmjopen.bmj.com/site/about/guidelines.xhtml

Outcome variables

The primary outcomes were: 1) the first point of care for an episode of illness, 2) healthcare-seeking pathway demonstrated the movement of the caregivers from one healthcare option to another for seeking healthcare beginning with home care or traditional healers and extending to informal or formal health care services, following an episode of illness in a child and 3) antibiotic prescribing for an episodes of illness where some kind of treatment was provided to a sick child. The secondary outcomes were to determine the association of the socio-demographic factors related to the sick child and child's caregiver with the HSB.

Data analysis

Data were converted from the paper HSB diaries to an electronic format using Microsoft .NET Framework 4 (Microsoft Corp., Redmond, WA, USA). Stata 14.1 (Stata Corp. College Station, TX, USA) and IBM SPSS Statistics 23.0 (IBM Corp., Armonk, NY, USA) were used for data management and analysis.

Descriptive statistics was used to portray the demographic characteristics of the caregivers and children. The incidence of the different types of childhood illnesses and the HSB of the caregivers of the U-5 children for the recorded illnesses were calculated. Furthermore, the incidences of the different types of childhood illnesses were examined over four seasons. The seasons were divided as follows: pre-monsoon season (March to May), monsoon season (June to September), post-monsoon season (October to December) and winter season (January and February), which have average maximum temperatures of 37.5 ºC, 30 ºC, 29 ºC and 22 ºC, respectively [29]. The outcome of the analysis for the primary outcome was the first point of care for an episode of illness and the proportion of illness episodes receiving an antibiotic prescription

For peer review only - http://bmjopen.bmj.com/site/about/guidelines.xhtml

and was not based on the individual child. The first point of care was the caregiver's HSB from the onset of a child's illness to the first healthcare service approached by the caregiver in seeking treatment for the illness. This HSB was characterized as: 1) no treatment given, 2) home treatment (i.e., episodes of illness for which caregivers provided children home-based treatments, leftover medicines or medicines from local shops without consulting recognised healthcare providers), 3) healthcare-seeking from formal healthcare providers (i.e., episodes of illness where caregivers consulted formal healthcare practitioners)and 4) healthcare-seeking from IHCPs (i.e., episodes of illness where caregivers went to IHCPs for treatment). A mixed-effects logistic regression model accounting for two levels of clusters (children belonging to the same family and therefore having the same caregiver and the same children who had several episodes of a sickness during the follow-up period) was used to estimate the effects of children and caregiver characteristics on caregiver HSB over time. The percentage of illness episodes with an RTI (69%) was higher than that of the other common illnesses (GI infections-8%, fever-7%, skin infection-6% and others-10%) **(Table 1)**. Since, the number of episodes of RTI was greater than that of any other illness; the factors associated with the HSB of caregivers are presented only for RTI, whereas for all other complaints, the proportions of different HSBs are presented **(Table 1)**. The antibiotic prescription patterns for different episodes of illness were explored and analysed. Each prescribed antibiotic was coded according to the World Health Organization (WHO) Collaborating Centre for Drug Statistics Methodology, Anatomical Therapeutic Chemical (ATC) classification with the defined daily dose according to the fifth level of the ATC classification, J01 (antibacterial for systemic use) [30]. Antibiotic prescriptions were presented as the proportion of illness episodes receiving an antibiotic prescription and the number of antibiotic courses. The frequency and percentage were calculated for the categorical variables.

For peer review only - http://bmjopen.bmj.com/site/about/guidelines.xhtml

259 A mixed-effects multinomial stepwise logistic regression model was used to examine the association of the independent risk factors responsible for HSB, formal healthcare providers was taken as the base outcome. The independent risk factors were compared between getting no treatment, receiving home treatment and obtaining treatment from IHCPs (multinomial outcome variable), and the adjusted relative risk (aRR) along with the associated 95% confidence intervals (CI) and *p* values were reported.

265 **Ethical considerations**

266 All methods were carried out in accordance with the ethical standards set by the institutional ethics committee. An oral informed consent was obtained from all participating caregivers and from parents or guardians on behalf of child participants in the local language, Hindi.

269 Participating parents/guardians were explained about the study purposes and procedures orally and textually in the local language (Hindi). Parents/guardians were informed that they could withdraw from the study at any time. The identities of participating parents/guardians and children were masked using unique identifiers designed for the study, and no private demographic information was shared publicly. Furthermore, all healthcare providers' data collected from parents/guardians was kept non-identifiable. Additional assistance was provided to children in need of medical attention during follow-up by referral to nearby formal health facilities. Health camps were also organized after the study for all the children living in the study villages.

278 **Patient and public involvement**

279 Patients and the public were not involved in the design or planning of the study.

For peer review only - http://bmjopen.bmj.com/site/about/guidelines.xhtml

RESULTS

Out of a total possible 61,020 follow-up time points of HSB for 270 children available throughout the study period, 792 time points were lost to follow up due to the unavailability of the children (travelling) and death of one child **(Additional File 4)**. The median follow-up time points per selected child were 223 time points (Inter Quartile Range 220–225). The overall response rate for the study was 99%.

Socio-demographic characteristics of caregivers and children

Table 2 summarizes the socio-demographic characteristics of the children and their caregivers. The mean (±standard deviation [SD]) age of the children was 1.8±1.4years. All of the primary caregivers of the 270 children in the study were mothers. The mean (±SD) age of mothers was 24.8±5.21 years and ranged from 14 to 50 years. On average, each mother had two children. Half of the mothers had some school education, and 71% were homemakers. Of the mothers, 62% were from the households of families with more than five members and half were from households of poor socio-economic status.

Illness episodes, illness types and seasonal variations in the number of illness episodes

During the study period, a total of 2,161 illness episodes were recorded in the 270 children. The average (±SD) number of illness episodes recorded per child was 8±5 episodes, (range1 to 29). The average (±SD) duration for which an episode of illness persisted was 4.4±3.7 days, (range1 to 63 days). The commonest illness reported in the study was RTIs (69%, **Table 1**).

For peer review only - http://bmjopen.bmj.com/site/about/guidelines.xhtml

A peak in the number of illness episodes was observed in the winter season (January-February 2015) during the 26th week of follow-up, which recorded 41 illness episodes. Another peak was observed in the second post-monsoon season (October-December 2014) during the 65th week with 36 illness episodes **(Figure 2)**.

Healthcare-seeking behaviours of caregivers and the mapping of the healthcare services approached for the treatment of illnesses in children

The caregivers of the children sought care from two types of healthcare providers: IHCPs and formal healthcare providers, and they also preferred home care for a child's illness. For 33% of episodes (n = 711), no treatment was offered to the child, most commonly for RTI (n = 92%, 654/711). In 86% (n = 607/711) of the illness episodes where no care/treatment was sought, the mothers believed that the illnesses were mild and self-limiting **(Table 1)**.

Of the remaining episodes (n = 1450) in which some healthcare was provided for the illness, most mothers approached IHCPs for treatment, accounting for 73% (n = 1062/1450) of these cases. The distribution of the different types of IHCPs from where the treatments were sought was as follows: informal private practitioners (90%, n = 962/1062), local drug vendors (7%, n=78/1062) and traditional healers (3%, n =22/1062). Formal health care providers were approached for 5% (n = 72/1450) of illness episodes, and the different types included formal private practitioners (44%, n=32/72), public/private healthcare facilities (24%, n=17/72), lady health visitors (26%, n=19/72) and treatment providers at health camps conducted by medical colleges (6%, n=4/72). The second most common choice for child's illness was giving home care (22%, n=316/1,450; **Table 1** and **Figure 3**). Home care included providing medicines in 67% (n = 211/316, which included leftover medicines from

previous prescriptions and getting medicines prescribed in old prescription from local general/medical store) and homemade remedies in 35% (n = 105/316, which included herbal tea, herbal oil, herbal paste made of cloves and honey).

Of those illness episodes where care was sought, in 72% (n = 1045/1450) illness episodes mothers provided care on the same day as the start of the illness **(Table 1)**. The main reasons for a delay of more than 24 hours in seeking healthcare were: lack of money, lack of time and no transport facility available at the time of illness.

Healthcare-seeking pathways

A conceptual framework of the healthcare-seeking pathways was developed using the information from the HSB diaries. Figure 3 describes the overall healthcare-seeking pathways of the caregivers of the children during the episodes of illness irrespective of the type of illness. The dashed arrows in Figure3 show the HSB of the mothers from the onset of illness to the first point of care. The pathways demonstrate that IHCPs were the most preferred first point of care and accounted for 73% (n = 1062/1450) of the cases in which a child received treatment **(Figure3)**. The continuous arrows in Figure 3 represent the change in healthcare-seeking pathways between: home care, IHCPs and formal healthcare providers. Some caregivers appeared to switch between multiple healthcare facilities or providers (8%, n = 118/1450), especially when the illnesses lasted for more than one week. The most common pathway taken was to switch from one IHCP to another. In 4% (n = 52/1450) of the illness episodes regardless of the illness duration, treatment at home or by a traditional healer was performed along with allopathic therapy.

Mixed-effects multinomial logistic regression results for the effect of socio-demographic factors on the healthcare-seeking behaviour of caregivers

For peer review only - http://bmjopen.bmj.com/site/about/guidelines.xhtml

The risk factors for HSB were determined by mixed-effects multinomial logistic regression with treatment from formal healthcare providers as a base outcome, as seen in Table 3. Compared to formal healthcare the aRR for getting no treatment, receiving home care and obtaining treatment from IHCPs was higher when the type of illness was RTI as compared to the other illnesses reported (aRR = 11.54, 95% CI 6.14-21.71; 1.82, 95% CI 1.03-3.24 and 1.29, 95% CI 0.75-2.23 respectively). Compared to formal healthcare the aRR for getting no treatment, receiving home care and obtaining treatment from IHCPs was higher among illiterate mothers as compared to educated mothers (aRR = 2.86, 95% CI 1.41-5.80; 2.38, 95% CI 1.21-4.68 and 1.93, 95% CI 0.90-3.74 respectively), and also compared to formal healthcare the aRR for getting no treatment, receiving home care and obtaining treatment from IHCPs was higher when the mother was a home maker as compared to working mothers (aRR = 2.90, 95% CI 1.43-5.90; 4.17, 95% CI 2.06-8.46 and 2.10, 95% CI 1.16-3.79 respectively). Socio-economic status was associated with HSB across all quintiles, with the highest aRR for no treatment in the two lowest socio-economic quintiles (aRR = 6.59, 95% CI 2.15-20.23 and 6.39, 95% CI 2.13-18.86 respectively), as seen in Table 3.

Antibiotic prescribing for common childhood illnesses

Out of a total of 1450 illness episodes, 46% (n=670) received antibiotics. In a total of 670 episodes where antibiotics were given, a total of 807 antibiotic courses were prescribed and administered as single course of antibiotics (69%, n = 559/807) and up to a total of four antibiotics (14%, n = 111/807). Parenteral formulations (3%, n = 21/807) were prescribed less often than oral formulations (88%, n = 713/807), remaining includes topical formulations (9%, n = 73/807). In total, 85% (n = 694/807) of the administered antibiotic courses were prescribed by IHCPs. The majority (85%, n = 572/670) of antibiotics prescribed were broad spectrum and the

most commonly prescribed were third-generation cephalosporin (J01DD) (n=259, 39%) followed by penicillin with an extended spectrum (J01CA) (n=94, 14%) and fluoroquinolones (J01MA) (n=86, 13%) **(Table 4)**. Antibiotics were prescribed more frequently in cases of skin infections (62%) and GI infections (45%) as compared to fever alone (35%) **(Table 1)**.

DISCUSSION

This is the first study to prospectively follow the HSB of caregivers of a cohort of U-5 children treating common childhood illnesses and the antibiotic prescribing during the treatment in a community setting over a period of 2 years. This study explored healthcare-seeking pathways and antibiotic prescribing for acute childhood illnesses and investigated the socio-demographic determinants of caregivers affecting the HSB during an event of acute illness in a child.

The findings highlight important information about rural HSB during common childhood illnesses in India. The healthcare-seeking pathway differed considerably among caregivers because of interplay of a variety of factors. For example, India's wealthier classes tend to seek healthcare from multiple providers, whereas the poorer classes often get no treatment at all [19, 31]. The different points of care approached by the caregivers were: informal private practitioners, local drug vendors, traditional healers, formal private practitioners, public/private healthcare facilities, lady health visitors and providers of treatment at health camps conducted by medical colleges. Each caregiver visited at least one of the above listed healthcare service providers once or more for a variety of reasons during the study period. IHCPs were the most frequent service providers for first and second point of healthcare visits. Only 5% of the mothers took their child to formal healthcare providers. In our study informal private practitioners (66%) were the most greatly preferred choice of caregivers when seeking healthcare despite existing

389 formal healthcare facilities in the nearby vicinity **(Figure 3)**. The result contrast with those of
390 other studies from India that have reported that government facilities were approached more
391 often [32, 33]. However, private providers were also found to be preferred in India's state of
392 West Bengal [34]. Public and private healthcare systems coexist in almost all countries with
393 limited resources. India has one of the most privatized health systems in the world [35]. The
394 reason why private healthcare providers are generally preferred is due to their ease of access and
395 the inability of public healthcare systems to provide access to quality care [36]. Public hospitals
396 in India have had issues related to low quality treatments, long waiting periods, inadequate
397 infrastructure and doctor shortages [37]. Most administrative authorities are indifferent to
398 educating IHCPs and encourage them to work illegally as IHCPs address the needs unmet by the
399 formal healthcare system [38, 39].

400 In our study, RTIs were the most commonly occurring illnesses reported in U-5 children, which
401 is unlike previous cross-sectional studies that have reported greater incidence of diarrhoeal cases
402 as compared to respiratory infection [33]. This may be attributed to Swachh Bharat
403 Mission (SBM)/Swachh Bharat Abhiyan (SBA)/Clean India Mission a country-wide campaign
404 initiated by the government of India in 2014 [40]. The impact assessment study of India's
405 Swachh Bharat Mission on acute diarrheal disease outbreaks analysed acute diarrheal diseases
406 from 2010 to 2018 and reported a decline in the pattern of acute diarrheal disease outbreaks
407 during the intervention period between 2014-2018 [41, 42]. Increased numbers of illness
408 episodes were noted in the monsoon and post-monsoon seasons (June–December), varying
409 slightly from the results of other studies. This may be attributable to different climate patterns
410 among regions and varied season definitions over time for a region **[29, 43]**.

For peer review only - http://bmjopen.bmj.com/site/about/guidelines.xhtml

The present study also examined the effect of socio-demographic factors on the HSB of caregivers. The results from our study show that socio-demographic factors, such as the mother's education, occupation and socio-economic status were associated with where the caregivers sought healthcare and similar findings have also been reported in other studies [34, 44]. Maternal education levels and occupations are significant HSB determinants [34]. This may be due to the fact that educated mothers are more likely to have a better understanding of healthcare and provide preventive and curative services for the child. A mother's education is associated with WASH (water, sanitation and hygiene) and with HSBs relevant to decreasing childhood mortality [45, 46].

Overall, the antibiotic prescription rate found in our study was 46%. Fewer antibiotics were prescribed in this study than has been reported in other studies [8-10]. This may be due to the fact that the majority of the illnesses reported in this study were RTIs where no treatment was given to the child in 44% of the episodes and home care was provided in 13%, suggesting that people in rural areas have some understanding about illness severity. Nevertheless, in our study results, 24% of the RTIs were cases of simple colds and coughs where antibiotics were prescribed, out of which 89% of the prescriptions were from IHCPs [5]. This implies inappropriate antibiotic prescribing according to the Indian National Treatment Guidelines for Antimicrobial Use in Infectious Diseases, which recommends antibiotics only for the treatment of pneumonia and tonsillitis, classified as RTIs, and for dysentery, classified under GI infections. Also 42% (n=335/807) of the total antibiotic course prescribed were of the "WATCH group antibiotics" classified under WHO AWaRe classification of antibiotics 2019. The classification was to emphasize the importance of optimal uses of antimicrobials and potential for antimicrobial resistance. WATCH group antibiotics includes antibiotics that have higher

For peer review only - http://bmjopen.bmj.com/site/about/guidelines.xhtml

resistance potential and includes most of the highest priority agents among the Critically Important Antimicrobials for Human Medicine and/or antibiotics [47]. It has been stated that the healthcare in rural areas in India is predominantly served by informal healthcare providers, who have been reported as often prescribing antibiotics for common illnesses inappropriately [8, 26].

The longitudinal follow-up of caregiver HSB in the same cohort helped us to generate a detailed picture of healthcare-seeking pathways. This allowed us to understand several aspects of these pathways, including delays providing treatment, variability in the caregiver pathways and factors affecting healthcare-seeking pathway. Repeated recordings of the HSB occasions from the same group of caregivers also increased reliability. In addition, repeated weekly data collection from the same group of caregivers could control the factors that cause variability between responses. Furthermore, the recall period of 2 days reduced the chances of recall bias. Information on the episodes of illness was provided by the mothers and thus was subjective in the sense that none of the episodes of illness were validated externally by any medical examination. But the classification of the sickness was performed by the trained research assistants, as evidence suggests that caregivers may be unable to accurately classify illnesses [48]. The quality of data is good and reliable. Providers co-operated in providing the details of the medicines dispensed loose as they happen to remember the medicine whether or not they keep any records for the same, still some of the medicines were not identifiable.

Conclusions

In our rural cohort for many acute episodes of illnesses no treatment or home treatment was done, which resulted in overall reduced antibiotic prescribing. The commonest pathway taken for seeking healthcare was visiting IHCPs which is suggestive of the fact that they are major

healthcare providers in rural areas. Most of the antibiotics were prescribed by IHCPs, and were commonly prescribed for illnesses where they were not indicated.

Recommendations

Effective awareness and health education programmes at community level should be introduced to cater to illiterate and low-income populations to improve their healthcare-seeking practices. IHCPs should be trained in order to integrate them into the legal healthcare system, thus strengthening the healthcare system, especially in rural areas. This can be done under the Prime Minister skill development program [49]. A pilot program in West Bengal has shown encouraging results [39].

ETHICS STATEMENT

Patient consent for publication

Not required.

Ethical approval

The study was approved by the Institutional Ethics Committee of the RDGMC, Ujjain, India (No: 2013/07/17-311).

ACKNOWLEDGEMENT

The authors thank the participating caregivers for providing their valuable time. We would also like to thank Medical Director, Dr.V.K. Mahadik and the management of R.D. Gardi Medical College, Ujjain for providing administrative assistance for the study. We are also thankful to research assistants Sunita, Vikram, Bharat, Pooja, Maya, Mamta, Mukesh, Bhaarat for assisting

in data collection, project staff Priyank Soni, Devis Saha, Vivek Parashar for processing data and Ankit Garg and Giriraj Singh Sisodiya for database management.

DECLARATION OF INTEREST

None declared

AUTHOR CONTRIBUTIONS

VD together with MP, AP, MS, SK, AJT and CSL initiated the concept and formulated the initial design of the study. SK and VD are the main persons responsible for the study coordination. MP and MS provided the critical comments on the concept and design. SK and VD were responsible for data collection. SK performed data analysis. AP and GM supported the data analysis. SK drafted the first version of the manuscript and constructed all tables and designed all figures. AP and VD commented on the first draft. AJT was the senior advisor to the project. CSL is the principal investigator for the project. All authors have commented earlier versions of the manuscript and read and approved the final version of the manuscript.

FUNDING

The project was funded by the Swedish Research Council (grant no 521-2012-2889). The funders had no role in study design, data collection and analysis, decision to publish, or preparation of the manuscript.

DATA AVAILABILITY

The datasets used and/or analysed during the current study are available from the corresponding author on reasonable request. Due to ethical and legal restrictions, all inquiries should be made with The Chairman, Ethics Committee, R.D. Gardi Medical College, Agar Road, Ujjain, India

456006 (Emails: iecrdgmc@yahoo.in, uctharc@sancharnet.in), giving all details of the publication. For reference, please quote ethical permission no. 311, dated July 17, 2013.

FIGURES

Figure 1: Map showing geographical location of the study villages, formal/informal healthcare services within the 10 Km distance from the central village in Ujjain district, MP, India.

Figure 2: Distribution of total number of the episodes of illness recorded over the period of 113 weeks (with corresponding seasons)

Figure 3: Healthcare-seeking pathway of the caregivers of under-5 children, under study in rural Ujjain, India.

REFERENCES

1. **World Health Organization. Pre-Antibiotic era looming large - The world is almost out of time. 2014. http://www.searo.who.int/mediacentre/features/2014/rd-statement-amr2014/en/. Accessed on 2 Dec 2020**.
2. Levy SB, Marshall B: **Antibacterial resistance worldwide: causes, challenges and responses**. *Nat Med* 2004, **10**(12 Suppl):S122-129.
3. Meek RW, Vyas H, Piddock LJW: **Nonmedical Uses of Antibiotics: Time to Restrict Their Use?** *PLoS Biol* 2015, **13**(10):e1002266-e1002266.
4. **Child Global Burden of Disease, Adolescent Health, Reiner RC,Jr., Olsen HE, Ikeda CT, Echko MM, Ballestreros KE, Manguerra H, Martopullo I, Millear A, et al: Diseases, Injuries, and Risk Factors in Child and Adolescent Health, 1990 to 2017: Findings From the Global Burden of Diseases, Injuries, and Risk Factors 2017 Study**. *JAMA pediatrics* 2019, **173**(6):e190337.
5. **National treatment guidelines for antimicrobial use in infectious diseases. National center for disease control , Directorate General of Health Services, Ministry of Health & Family Welfare. Government of India 2016. http://pbhealth.gov.in/AMR_guideline7001495889.pdf. Accessed on 2 Dec 2020**.
6. Pathak A, Upadhayay R, Mathur A, Rathi S, Lundborg CS: **Incidence, clinical profile, and risk factors for serious bacterial infections in children hospitalized with fever in Ujjain, India**. *BMC infectious diseases* 2020, **20**(1):162.
7. Pathak D, Pathak A, Marrone G, et al: **Adherence to treatment guidelines for acute diarrhoea in children up to 12 years in Ujjain, India--a cross-sectional prescription analysis**. *BMC infectious diseases* 2011, **11**:32.

8. Khare S, Purohit M, Sharma M, Tamhankar AJ, Lundborg CS, Diwan V, Pathak A: **Antibiotic Prescribing by Informal Healthcare Providers for Common Illnesses: A Repeated Cross-Sectional Study in Rural India**. *Antibiotics (Basel, Switzerland)* 2019, **8**(3).
9. Pathak A, Mahadik K, Dhaneria SP, et al: **Antibiotic prescribing in outpatients: Hospital and seasonal variations in Ujjain, India**. *Scandinavian journal of infectious diseases* 2011, **43**(6-7):479-488.
10. Sharma M, Damlin A, Pathak A, et al: **Antibiotic Prescribing among Pediatric Inpatients with Potential Infections in Two Private Sector Hospitals in Central India**. *PloS one* 2015, **10**(11):e0142317.
11. Costelloe C, Metcalfe C, Lovering A, et al: **Effect of antibiotic prescribing in primary care on antimicrobial resistance in individual patients: systematic review and meta-analysis**. *BMJ (Clinical research ed)* 2010, **340**:c2096.
12. Miller R, Goodman C: **Performance of retail pharmacies in low- and middle-income Asian settings: a systematic review**. *Health policy and planning* 2016, **31**(7):940-953.
13. Rao M, Rao KD, Kumar AK, et al: **Human resources for health in India**. *Lancet* 2011, **377**(9765):587-598.
14. **World Health Organization. Improving the containment of antimicrobial resistance. WHA. https://www.searo.who.int/entity/medicines/topics/wha_58_27.pdf. 2005. Accessed on 2 Dec 2020**.
15. Dekker AR, Verheij TJ, van der Velden AW: **Inappropriate antibiotic prescription for respiratory tract indications: most prominent in adult patients**. *Family practice* 2015, **32**(4):401-407.
16. Viberg N, Kalala W, Mujinja P, Tomson G, Lundborg CS: **"Practical knowledge" and perceptions of antibiotics and antibiotic resistance among drugsellers in Tanzanian private drugstores**. *BMC infectious diseases* 2010, **10**.
17. Conner M, Norman P: **The theory of planned behaviour and health behaviours. Predicting Health Behaviours: Research and Practice with Social Cognition Models. Buckingham: Open University Press**. 1996:121-162.
18. **Registrar General of India (2012) Census of India 2011. Government of India, New Delhi; http://www.censusindia.gov.in/vital_statistics/SRS_Reports_2012.html**.
19. Barik D, Thorat A: **Issues of Unequal Access to Public Health in India**. *Front Public Health* 2015, **3**:245-245.
20. Wattal C, Goel N: **Tackling antibiotic resistance in India**. *Expert review of anti-infective therapy* 2014, **12**(12):1427-1440.
21. **Encyclopedia Britannica. Madhya Pradesh [Internet]. Britannica Academic. https://academic.eb.com/levels/collegiate/article/MAdhya-Pradesh/111205 (2017). Accessed on 2 Dec 2020**.
22. **National Family Health Survey 4. 2015 -16. State Fact Sheet Madhya Pradesh. http://rchiips.org/NFHS/pdf/NFHS4/MP_FactSheet.pdf. Accessed on 2 Dec 2020**.
23. **Subnational Human Development Index - Area Database - Global Data Lab. Institute for Management Research Radbound University. 2018. https://www.https://globaldatalab.org/shdi/shdi/. Accessed on 2 Dec 2020.**
24. Stalsby Lundborg C, Diwan V, Pathak A, et al: **Protocol: a 'One health' two year follow-up, mixed methods study on antibiotic resistance, focusing children under 5 and their environment in rural India**. *BMC Public Health* 2015, **15**:1321.
25. **World Health Organization. Classifying health workers: Mapping occupations to the international standard classification. https://www.who.int/hrh/statistics/Health_workers_classification.pdf. 2008. Accessed on 2 Dec 2020.**

For peer review only - http://bmjopen.bmj.com/site/about/guidelines.xhtml

26. Sudhinaraset M, Ingram M, Lofthouse HK, et al: **What is the role of informal healthcare providers in developing countries? A systematic review**. *PloS one* 2013, **8**(2):e54978.
27. Rowe AK, Lama M, Onikpo F, et.al.: **Design effects and intraclass correlation coefficients from a health facility cluster survey in Benin**. *International journal for quality in health care : journal of the International Society for Quality in Health Care* 2002, **14**(6):521-523.
28. Vyas S, Kumaranayake L: **Constructing socio-economic status indices: how to use principal components analysis**. *Health policy and planning* 2006, **21**(6):459-468.
29. Arora SK, Jha B: **Indian Meteorological Department 2018. http://mausam.imd.gov.in/. Accessed on 2 Dec 2020**.
30. WHO: **World Health Organization Anatomical Therapeutic Chemical (ATC) classification system: guidelines for ATC classification and DDD assignment 2018. WHO Collaborating Centre for Drug Statistics Methodology, ATC classification index with DDDs, 2018. Oslo, Norway**. 2018.
31. Balarajan Y, Selvaraj S, Subramanian SV: **Health care and equity in India**. *Lancet* 2011, **377**(9764):505-515.
32. Chauhan RC, Manikandan., Purty AJ, et al: **Determinants of health care seeking behavior among rural population of a coastal area in South India**. *Int J Scientific Reports* 2015, **1**(2):118-122.
33. Kalita B, Borah MK, Kakati R, et al: **Primary caregivers health seeking behaviour for under-five children: A study in a rural block of Assam, India**. *Ntl J Community Med* 2016.
34. Kanungo S, Bhowmik K, Mahapatra T, et al: **Perceived morbidity, healthcare-seeking behavior and their determinants in a poor-resource setting: observation from India**. *PloS one* 2015, **10**(5):e0125865.
35. Mackintosh M, Channon A, Karan A, Selvaraj S, Cavagnero E, Zhao H: **What is the private sector? Understanding private provision in the health systems of low-income and middle-income countries**. *Lancet* 2016, **388**(10044):596-605.
36. Peters DH, Rao KS, Fryatt R: **Lumping and splitting: the health policy agenda in India**. *Health policy and planning* 2003, **18**(3):249-260.
37. Satpathy SK: **Public health infrastructure in rural India: challenges and opportunities**. *Indian journal of public health* 2005, **49**(2):57-62.
38. Chandra S, Bhattacharya S: **Unqualified Medical Practitioners: Their Illegal but Indispensable Role in Primary Healthcare. Economic and Political Weekly, 2019. Vol LIV No. 5 (February 2, 2019): p. 36-44**.
39. Das J, Chowdhury A, Hussam R, et.al.: **The impact of training informal health care providers in India: A randomized controlled trial**. *Science* 2016, **354**(6308):aaf7384.
40. **Swachh Bharat Mission - Gramin, Department of Drinking water & Sanitation, Ministry of Jal Shakti, Government of India. https://www.swachhbharatmission.gov.in/SBMCMS/study-survey.htm. Accessed on 24 Feb 2021**.
41. **Review of health data in selected ODF and non-ODF districts under the SBM (Report of Findings). Ministry of Drinking Water and Sanitation (MoDWS), Government of India. 2017. https://www.https://jalshakti-ddws.gov.in/sites/default/files/BMGF_Health_Impact_Study_final.pdf. Accessed on 24 Feb 2021**.
42. Dandabathula G, Bhardwaj P, Burra M, Rao PVVP, Rao SS: **Impact assessment of India's Swachh Bharat Mission - Clean India Campaign on acute diarrheal disease outbreaks: Yes, there is a positive change**. *Journal of family medicine and primary care* 2019, **8**(3):1202-1208.
43. Reddaih VP, Kapoor SK: **Epidemiology of pneumonia in rural underfives**. *Ind J Com Medicine* 1990, **57**:701-704.

For peer review only - http://bmjopen.bmj.com/site/about/guidelines.xhtml

44. Chandwani H, Pandor J: **Healthcare-seeking behaviour of mothers regarding their children in a tribal community of Gujarat, India**. *Electronic Physician* 2015, **7**(1):990-997.
45. **Rebecca YS. DHS Comparative reports: child morbidity and treatment patterns. Calverton, Maryland: ORC macro, Calvetron, USA, 2004.** .
46. **Inter-Agency and Expert Group on MDG indicators. The millennium development goals report 2012. New York: United Nations, 2012.**
47. **World Health Organisation. WHO releases the 2019 AWaRe Classification Antibiotics. 2019. https://www.who.int/medicines/news/2019/WHO_releases2019AWaRe_classification_antibiotics/en/. Accessed on 6 Apr 2020.**
48. Hazir T, Begum K, El Arifeen S, Khan AM, Huque MH, Kazmi N, Roy S, Abbasi S, Rahman QS, Theodoratou E *et al*: **Measuring coverage in MNCH: a prospective validation study in Pakistan and Bangladesh on measuring correct treatment of childhood pneumonia**. *PLoS medicine* 2013, **10**(5):e1001422.
49. India Go: **Schemes & Initiatives through NSDC. Pradhan Mantri Kaushal Vikas Yojana 2016-2020. Ministry of Skill Development and Entrepreneurship. https://www.msde.gov.in/schemes-initiatives/schemes-initiatives-through-nsdc#:~:text=The%20PMKK%20program%20provides%20financial,Government%20at%20the%20district%20level. Accessed on 23 Dec 2020**.

Table 1: Incidence and type of illness episodes; antibiotic prescribing and the first point of care recorded during healthcare-seeking behaviour follow up for the under-5 children in the study, in rural Ujjain

Variables	Total episodes N=2161	Healthcare-seeking behaviour				Illness episodes with antibiotics prescribed N=670
		No treatment N=711	Home care[(a)] N=316	Formal Healthcare[(b)] N=72	Informal Healthcare[(c)] N=1062	
**Illnesses reported**						
RTI^	1501	654 (44)	202 (13)	38 (3)	607 (40)	354 (24)
GIinfections^	182	8 (5)	31 (17)	17 (9)	126 (69)	82 (45)
Fever	147	4 (3)	28 (19)	4 (3)	111 (75)	52 (35)
Skin infections	138	14 (10)	12 (9)	7 (5)	105 (76)	86 (62)
Others	193	31 (16)	43 (22)	6 (3)	113 (59)	96 (50)
**Reasons for not taking treatment**						
Thought as illness is self-curable	-	333 (47)	-	-	-	-
Illness is mild	-	274 (39)	-	-	-	-
Others**	-	104 (14)	-	-	-	-
**Delay in seeking treatment (in days)**		-	**N=1450 (%)**			-
On the same day		-		1045 (72)		-
After one day		-		305 (21)		-
After two days		-		61 (4)		-
After three days		-		39 (3)		-

[a]Episodes of illness for which caregivers did not consult any healthcare provider and gave child home treatment (home remedies) or left over medicines or brought medicines from local shop on their own; [b] Episodes of illness for which caregiver consulted formal government/private practitioner, Government/private healthcare facility, community health visitor, Lady health visitor (ASHA/Anganwadi worker), Health camps; [c] Episodes of illness for which caregiver consulted informal private practitioner, local drug vendors without license, traditional healers; ^CI- Confidence Interval; SD- Standard Deviation; RTI- Respiratory Tract Infections; GI infections- Gastrointestinal infections

For peer review only - http://bmjopen.bmj.com/site/about/guidelines.xhtml

692 **Table 2: Socio-demographic characteristics of mothers (caregivers) and under-5 children**
693 **under study in rural Ujjain, India**
694

Variables	Frequency (n)	Percentage (%)
**Child's Age (in years)(N=270)**		
≤ 1	140	52
2	61	23
3	23	8
4	26	10
5	20	7
**Child's Gender(N=270)**		
Male	136	50.4
Female	134	49.6
**Mother's Age (in years)(N=141)**		
14-25	88	62
> 25	53	38
**Education of mother(N=141)**		
Illiterate	71	50.4
School education and above*	70	49.6
**Occupation of mother(N=141)**		
Home maker	101	71.6
Working**	40	28.4
**Family members/ household(N=118)**		
≤ 5	45	38
> 5	73	62
**Parity (number of children)(N=141)**		
1-2	95	67
≥ 3	46	33
**Socio-economic status (PCA)(N=118)**		
Fifth Quintile (Wealthiest)	17	14
Fourth quintile (Wealthier)	23	19
Third quintile (Middle)	28	24
Second quintile (Poorer)	21	18
Fist quintile (Poorest)	29	25

695 ^PCA- Principle component analysis; *School education includes primary school education from class 1 to class 8 and secondary
696 school education from class 9 to class 12; **Working includes Farmer, Labour worker, Job in public/private sector
697
698
699
700
701
702
703
704

Table 3: Mixed-effects multinomial logistic regression model for predictors of healthcare-seeking behaviour of caregivers for common illnesses reported in under-5 children in study in Ujjain, India

Base outcome: "Formal healthcare" for the illness reported during healthcare-seeking behaviour follow-up

Variable	No treatment given (n=711)		Home treatment (n=316)		Informal healthcare (n=1062)	
	aRR^ (95% CI)^	p value	aRR^ (95% CI)^	p value	aRR^ (95% CI)^	p value
**Illness Type**						
Other illness	1	-	1	-	1	-
RTI#	11.54 (6.14-21.71)	<0.001*	1.82 (1.03-3.24)	0.04*	1.29 (0.75-2.23)	0.36
**Education of mother**						
School education and aboveǂ	1	-	1	-	1	-
Illiterate	2.86 (1.41-5.80)	0.004*	2.38 (1.21-4.68)	0.01*	1.93 (0.90-3.74)	0.05
**Occupation of mother**						
Working**	1	-	1	-	1	-
Home maker	2.90 (1.43-5.90)	0.004*	4.17 (2.06-8.46)	<0.001*	2.10 (1.16-3.79)	0.02*
**Socio-economic status (PCA)**						
Fifth Quintile (Wealthiest)	1	-	1	-	1	-
Fourth quintile (Wealthier)	3.56 (1.30-9.77)	0.01*	3.24 (1.39-7.58)	0.007*	1.90 (0.81-4.45)	0.14
Third quintile (Middle)	3.51 (1.31-9.37)	0.01*	4.52 (1.72-11.87)	0.002*	3.20 (1.33-7.67)	0.01*
Second quintile (Poorer)	6.59 (2.15-20.23)	0.001*	9.20 (3.55-23.81)	<0.001*	4.38 (1.50-12.90)	0.01*
First quintile (Poorest)	6.39 (2.13-18.86)	0.001*	6.86 (2.62-18.01)	<0.001*	3.19 (1.23-8.26)	0.02*

^aRR: Adjusted Risk ratios, CI: Confidence Interval; *Significant p value <0.05; ǂ School education includes primary school education from class 1 to class 8 and secondary school education from class 9 to class 12; **Working includes Farmer, Labour worker, Job in public/private sector; n = number of yes response; #RTI: acute respiratory tract infection

Table 4: Number of illness episodes with antibiotic prescriptions categorised using anatomical therapeutic chemical (ATC) classification, reported during the healthcare-seeking behaviour follow-up in study in Ujjain, India

Illnesses	Penicillin with extended spectrum; amoxicillin and ampicillin with cloxacillin J01CA n= 94(%)	Penicillin with β -lactamase inhibitor, amoxicillin with clavulanic acid J01CR n= 59	Tetracyclines J01A n= 2	Cephalosporins; J01D			Sulfonamides and Trimethoprim J01E n= 50	Aminoglycosides J01GB n= 3	Macrolides J01FA n= 19	Fluoroquinolones J01MA n= 86	Metronidazole Nitazoxanide (P01AX11)P01 AB n= 12	Combinations of antibacterial J01RA n= 39
				1st generation J01DB n= 27	2nd generation J01DC n= 4	3rd generation J01DD n= 259						
RTI* (n=1501)	59(61)	33(56)	1(50)	18(67)	2(50)	173(67)	23(46)	1(67)	10(52)	33(38)	4(33)	18(46)
GI infections** (n=182)	8(9)	10(17)	-	3(11)	-	21(8)	1(2)	-	2(11)	15(18)	8(67)	15(38)
Fever (n=147)	9(10)	9(15)	-	3(11)	1(25)	23(9)	3(6)	-	3(16)	6(7)	-	2(5)
Skin infections (n=138)	10(10)	4(7)	1(50)	2(7)	-	15(6)	17(34)	-	3(16)	20(23)	-	3(8)
Others (n=193)	8(9)	3(5)	-	1(4)	1(25)	27(10)	6(12)	2(33)	1(5)	12(14)	-	1(3)

*** RTI- Respiratory tract infections, **GI infections- gastrointestinal infections; n- frequency of the respective category; Column percentages are corresponding to the column total; Row percentage will not match as more than one antibiotics were prescribed for the respective illness types in some prescriptions**

List of abbreviations

RTI - Acute respiratory tract infections

HSB - Healthcare-seeking behaviour

IHCPs - Informal healthcare providers

U-5 - Under-5

MP - Madhya Pradesh

DSS - Demographic surveillance site

RDGMC – Ruxmaniben Deepchand Gardi Medical College

GI - Gastro-intestinal

ATC - Anatomical Therapeutic Chemical Classification

aRR - Adjusted relative risk

CI - Confidence interval

SD - Standard deviation

aOR - Adjusted odds ratio

WHO – World Health Organization

For peer review only - http://bmjopen.bmj.com/site/about/guidelines.xhtml

Figure 1: Map showing geographical location of the study villages, formal/informal healthcare services within the 10 Km distance from the central village in Ujjain district, MP, India.

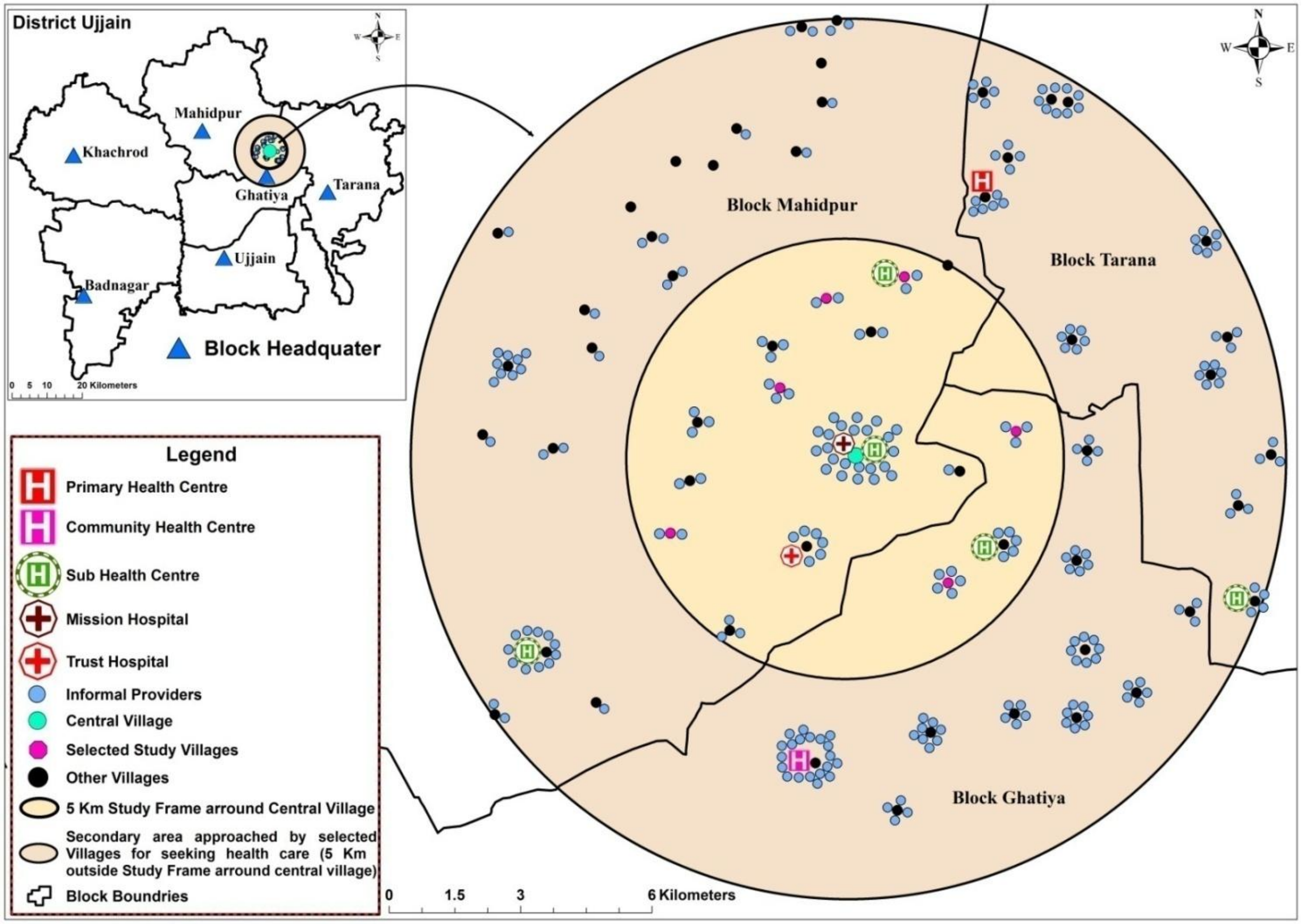

Map showing 5 Km study frame around the central village in Ujjain district, MP, India with the geographical location of the study villages, informal healthcare providers practising within the study frame and formal healthcare facilities located within the 10 Km distance from the central village within study frame in the inset map. (Source: Survey of India (SOI) in Dehra Dun, India (License No. BP11CDLA183)

Figure 2: Distribution of total number of the episodes of illness recorded over the period of 113 weeks (with corresponding seasons)

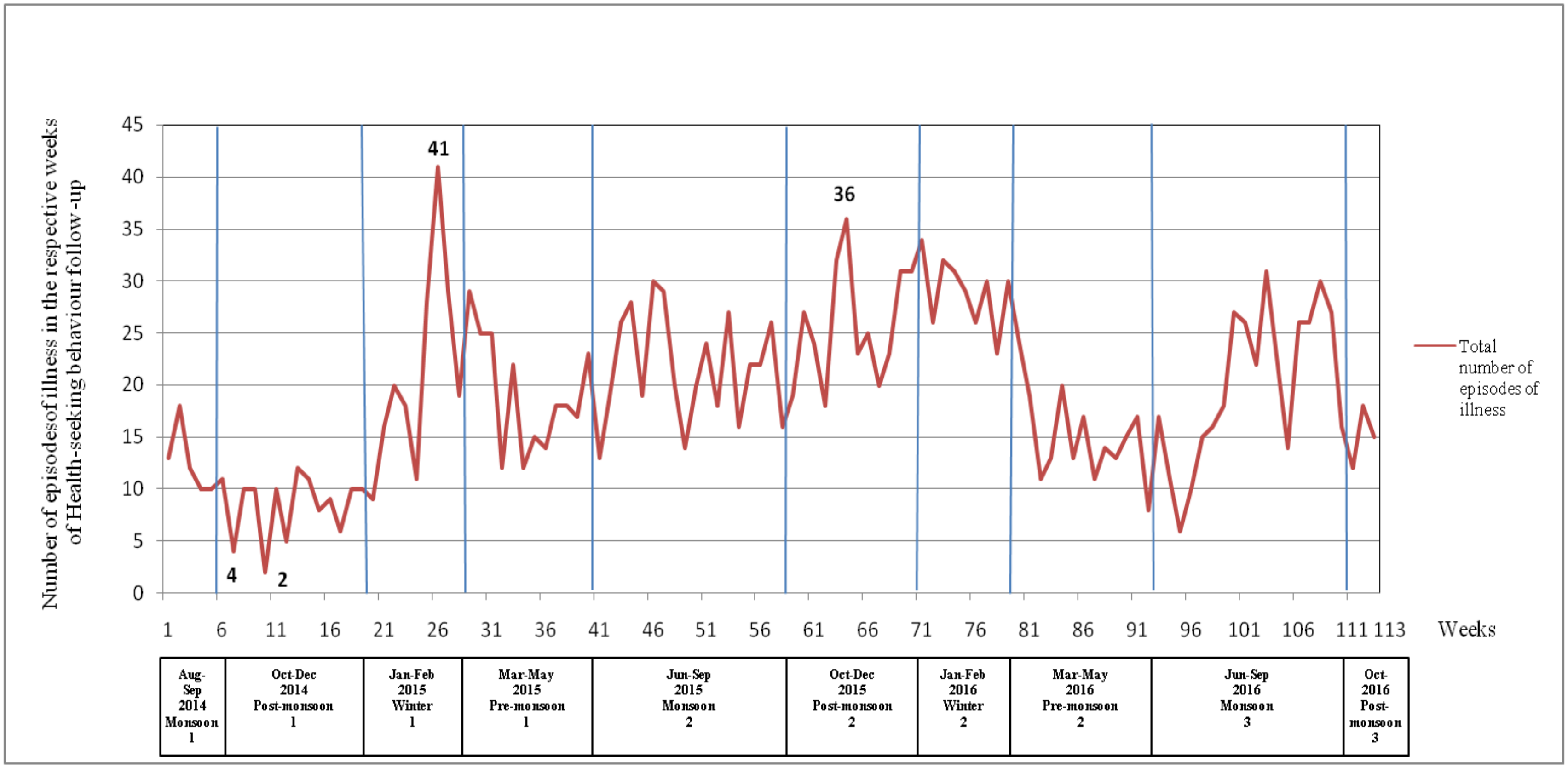

The X-axis represents the week from week 1 to week 113 (i.e, August 2014 to October 2016)with the corresponding seasons (August-September 2014 Monsoon 1, October-December 2014 Post-monsoon 1, January-February 2015 Winter 1, March-May 2015 Pre-monsoon 1, June-September 2015 Monsoon 2, October-December 2015 Post-monsoon 2, January-February 2016 Winter 2, March-May 2016 Pre-monsoon 2, June-September 2016 Monsoon 3, October 2016 Post-monsoon 3); Y-axis represents the number of episodes of illness reported during the healthcare-seeking behaviour follow-up in the respective week

Figure 3: Healthcare-seeking pathway of the caregivers of under-5 children, under study in rural Ujjain, India.

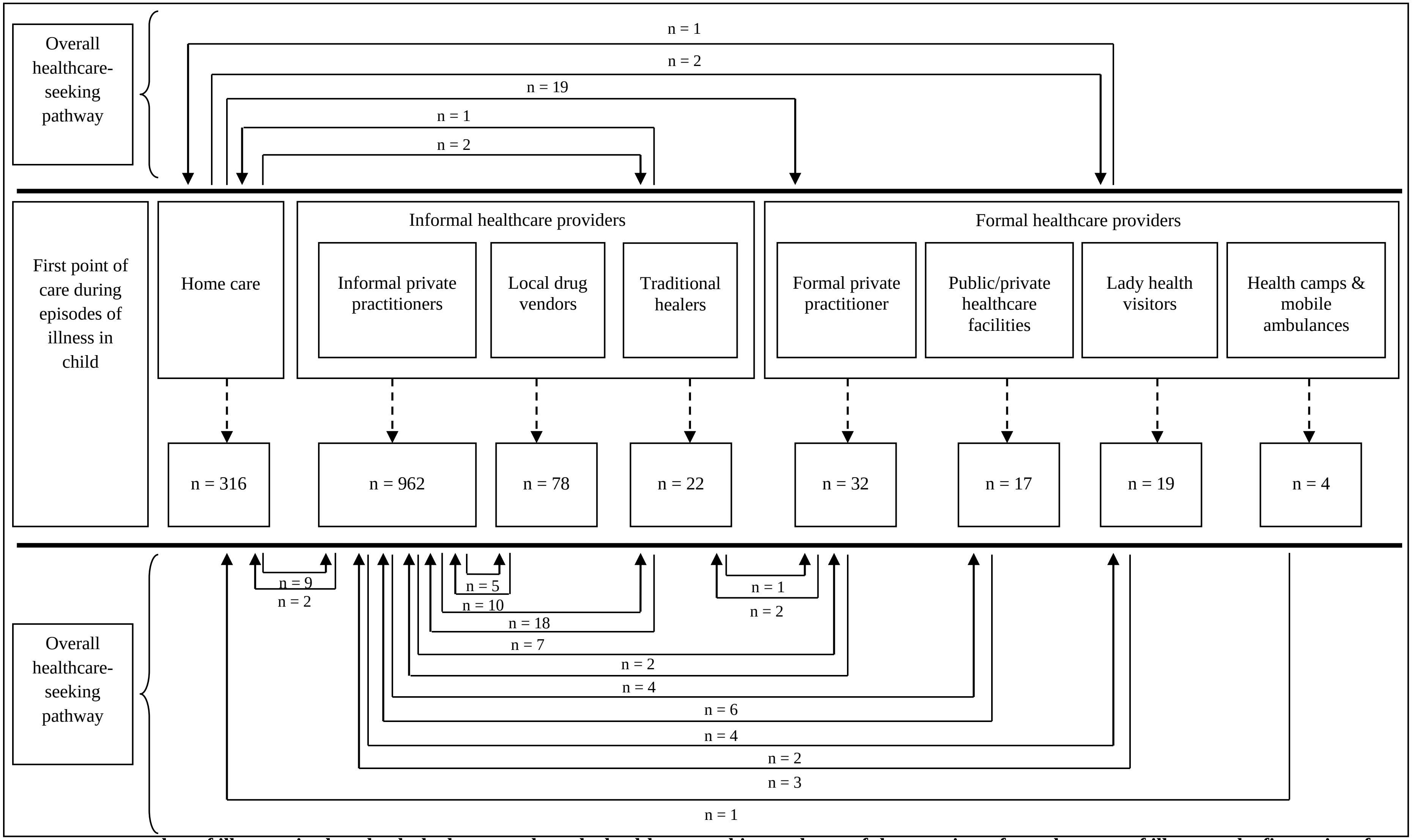

n= number of illness episodes; the dashed arrows show the healthcare-seeking pathway of the caregivers from the onset of illness to the first point of care; the continuous arrows represent the change in healthcare-seeking pathways between: home care, informal and formal healthcare providers.

Additional File 1: Socio-demographic survey questionnaire

Part1: Information on household members:

S.No.	Name	Sex (M/F)	Age	Relation with 'Head'	Educational Qualification	Occupation
1.						
2.						
3.						
4.						
5.						
6.						
7.						
8.						
9.						
10.						

Part2: Questionnaire

Gender of head of household
Respondent age
Gender of respondent
Family type
Number of family members
Number of children of age 0 to 1 year, 1 to 3 years, 0 to 5 years, 6 to 15 years
Number of members above 15 years of age
Type of caste
Which ration card does the family have
Type of house (kuccha, semi-kuccha, pucca)?
Do you own your house?
Number of rooms other than kitchen, toilet, storage
Do you pay bill for electricity
First priority source of electricity used
Second priority source of electricity used
Third priority source of electricity used
Fourth priority source of electricity used
Does your household possess:

- Radio
- Television
- Bicycle
- Motor cycle
- Bull/horse
- Car/jeep
- Tractor
- Harvester
- Generator

For peer review only

LPG
Water pump in house
water pump in field
Landline (Phone)
Mobile (Phone)
Internet
Cooler, table fan or Ceiling fan
Fridge
Diesel Engine
Livestock
Do you own any property and agriculture land?
If yes - How much property other than agriculture in square feet (sq.ft)?
If yes - How much is for agriculture in sq.ft?
First priority main source of drinking water
What kind of toilet facility do members of your household usually use?

For peer review only

Additional File 2: Healthcare-seeking behavior diary

S.NO.	QUESTIONS
	**Section A: General information about illness**
1.	History of presenting illness
2.	Did you seek any treatment for your child or gave anything on your own to your child for the illness?
3.	If **'NO'**, Why? **(end the interview)**
4.	If **'YES'**, Where?
	**Section B: Treatment at "HOME"**
5.	If 'HOME', describe the treatment?<br>**(if 'NO' skip to 12)**
6.	What medicine you gave your child? mention the type, dose and source **(ask for**
7.	Who advice you the above mentioned treatment for your child's illness?
8.	From where did you get the medicine **(over the counter etc)?**
9.	Estimated direct cost of home treatment **(in rupees)**
10.	Did your child got relief from the illness **(YES / NO)**
11.	If **'NO'**, where did you took your child next for the treatment
12.	Name of '**Healthcare facility/private practitioner/CHV/ LHV/Pharmacist** etc.' visited for
13.	Where the healthcare facility/ clinic are located?
14.	Why you took your child to this healthcare facility/ private practitioner?
15.	Mode of transport to reach the facility?
16.	Travel time **(in minutes)**
17.	Did they prescribe any medicine?
18.	If **'YES'** describe the medicine with the respective dose /provide prescription?
19.	Did you give your child the complete dose of prescribed medicine? **(YES/NO)**<br>**(if 'YES' skip to 21)**
20.	Why you did not gave the prescribed dose to your child?
21	Were there any adverse events from any of the medicines prescribed?
22.	Did your child have any relief from the illness?<br>**(YES / NO)**
23.	Were your child asked for any investigation? **(YES/NO)**<br>AndIf**"YES"** mention the type of investigation?<br>**(if 'NO' skip to 25)**
24.	Did you take your child for the investigations? **(YES/NO)**
25.	Estimated direct cost of treatment from healthcare facility / private practitioner<br>**(excluding the travel cost) (in rupees)**
26.	Transport Cost to reach healthcare facility/ private practitioner
	**Section D: Treatment by "Traditional healers"**
27.	Did you seek any treatment from the Traditional healers for your child's illness? **(YES/NO)**
28.	What kind of treatment was provided by them? Describe treatment
29.	Did your child got any relief from the illness? **(YES/NO)**
30.	Where do you generally seek the treatment for your child when he/she gets common cold, cough, fever, body ache?

For peer review only

Additional File 3: Presenting illness classification

**Presenting illness classification**	**Key illnesses under presenting illness classification**
RTI*	Cold,cough with/without fever,ear infection, tonsillitis
GI infections*	Diarrhoea with/without stomach pain, appetite loss vomiting, gas, constipation
Fever	Fever without other illness
Others	Illnesses that occurred rarely e.g. chicken pox, ring worm, injury, insect/animal bite, oral problems

* RTI- Acute respiratory tract infections, GI infections- Gastrointestinal infections

For peer review only

Additional File 4: Flow chart describing sample population, episodes of illness recorded among study population, under study in rural Ujjain, India.

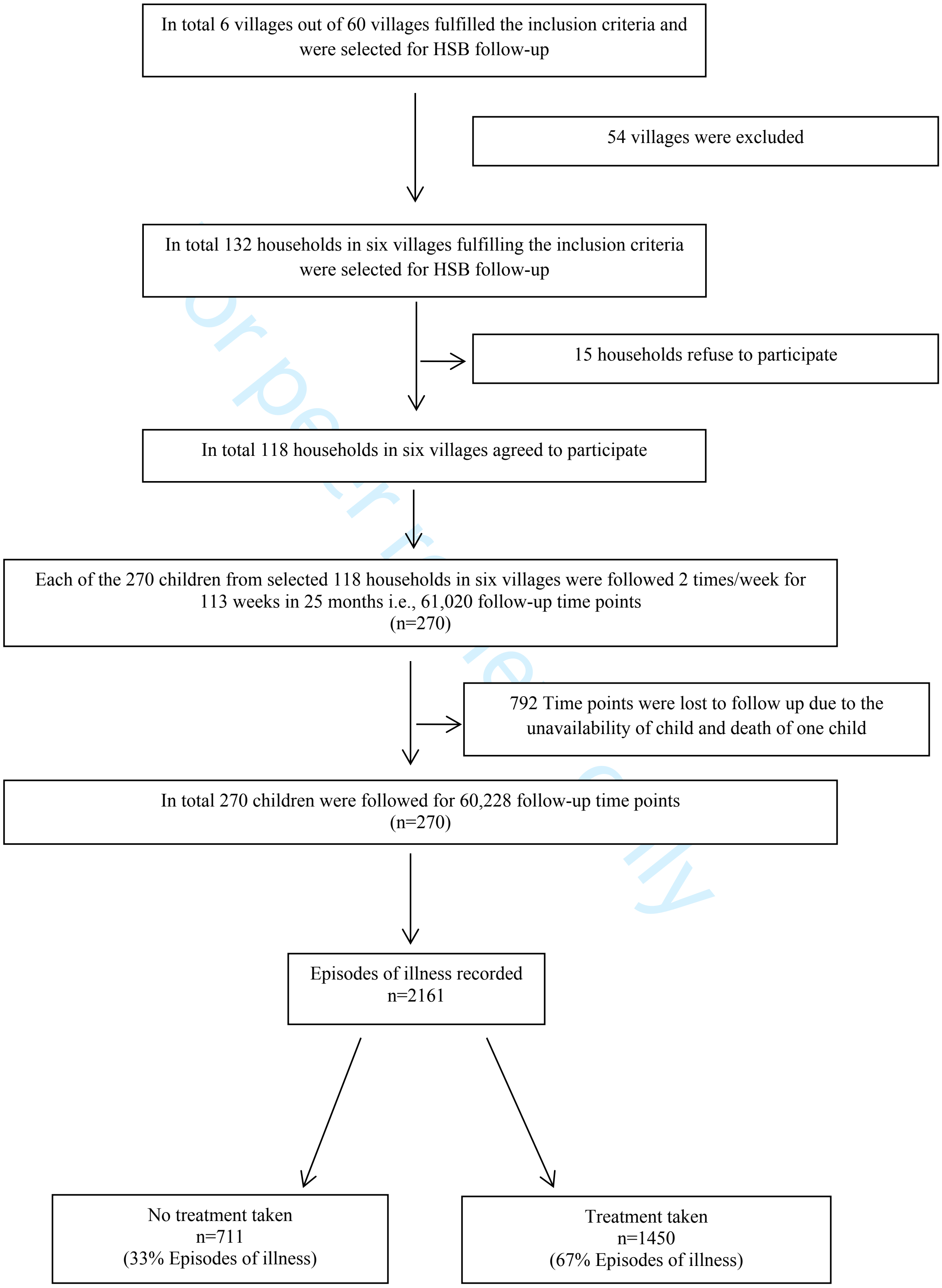

For peer review only

STROBE Statement—Checklist of items that should be included in reports of ***cohort studies***

	Item No	Recommendation	Page No
**Title and abstract**	1	(*a*) Indicate the study's design with a commonly used term in the title or the abstract	1
		(*b*) Provide in the abstract an informative and balanced summary of what was done and what was found	2-4
**Introduction**			
Background/rationale	2	Explain the scientific background and rationale for the investigation being reported	5-6
Objectives	3	State specific objectives, including any prespecified hypotheses	6
**Methods**			
Study design	4	Present key elements of study design early in the paper	6-7
Setting	5	Describe the setting, locations, and relevant dates, including periods of recruitment, exposure, follow-up, and data collection	7-10
Participants	6	(*a*) Give the eligibility criteria, and the sources and methods of selection of participants. Describe methods of follow-up	8-10
		(*b*)For matched studies, give matching criteria and number of exposed and unexposed	Not applicable
Variables	7	Clearly define all outcomes, exposures, predictors, potential confounders, and effect modifiers. Give diagnostic criteria, if applicable	10-11
Data sources/ measurement	8*	For each variable of interest, give sources of data and details of methods of assessment (measurement). Describe comparability of assessment methods if there is more than one group	11-13
Bias	9	Describe any efforts to address potential sources of bias	21
Study size	10	Explain how the study size was arrived at	8
Quantitative variables	11	Explain how quantitative variables were handled in the analyses. If applicable, describe which groupings were chosen and why	10-13
Statistical methods	12	(*a*) Describe all statistical methods, including those used to control for confounding	13
		(*b*) Describe any methods used to examine subgroups and interactions	12
		(*c*) Explain how missing data were addressed	10
		(*d*) If applicable, explain how loss to follow-up was addressed	14
		(*e*) Describe any sensitivity analyses	Not applicable
**Results**			
Participants	13*	(a) Report numbers of individuals at each stage of study—eg numbers potentially eligible, examined for eligibility, confirmed eligible, included in the study, completing follow-up, and analysed	14
		(b) Give reasons for non-participation at each stage	14
		(c) Consider use of a flow diagram	14
Descriptive data	14*	(a) Give characteristics of study participants (eg demographic, clinical, social) and information on exposures and potential confounders	14
		(b) Indicate number of participants with missing data for each variable of interest	14
		(c) Summarise follow-up time (eg, average and total amount)	14
Outcome data	15*	Report numbers of outcome events or summary measures over time	15-16

For peer review only

Main results	16	(*a*) Give unadjusted estimates and, if applicable, confounder-adjusted estimates and their precision (eg, 95% confidence interval). Make clear which confounders were adjusted for and why they were included	17
		(*b*) Report category boundaries when continuous variables were categorized	17
		(*c*) If relevant, consider translating estimates of relative risk into absolute risk for a meaningful time period	17
Other analyses	17	Report other analyses done—eg analyses of subgroups and interactions, and sensitivity analyses	Not applicable
**Discussion**			
Key results	18	Summarise key results with reference to study objectives	18-21
Limitations	19	Discuss limitations of the study, taking into account sources of potential bias or imprecision. Discuss both direction and magnitude of any potential bias	20-21
Interpretation	20	Give a cautious overall interpretation of results considering objectives, limitations, multiplicity of analyses, results from similar studies, and other relevant evidence	21-22
Generalisability	21	Discuss the generalisability (external validity) of the study results	Not applicable
**Other information**			
Funding	22	Give the source of funding and the role of the funders for the present study and, if applicable, for the original study on which the present article is based	23

*Give information separately for exposed and unexposed groups.

Note: An Explanation and Elaboration article discusses each checklist item and gives methodological background and published examples of transparent reporting. The STROBE checklist is best used in conjunction with this article (freely available on the Web sites of PLoS Medicine at http://www.plosmedicine.org/, Annals of Internal Medicine at http://www.annals.org/, and Epidemiology at http://www.epidem.com/). Information on the STROBE Initiative is available at http://www.strobe-statement.org.

For peer review only
